# High-resolution CMIP6 climate projections for Ethiopia using the gridded statistical downscaling method

**DOI:** 10.1038/s41597-023-02337-2

**Published:** 2023-07-12

**Authors:** Fasil M. Rettie, Sebastian Gayler, Tobias K. D. Weber, Kindie Tesfaye, Thilo Streck

**Affiliations:** 1 Biogeophysics, Institute of Soil Science and Land Evaluation, Hohenheim University, 70599 Stuttgart, Germany; 2 Ethiopian Institute of Agricultural Research (EIAR), Melkasa, Ethiopia; 3 Soil Science Section, Faculty of Organic Agricultural Sciences, University of Kassel, Kassel, Germany; 4 International Maize and Wheat Improvement Center (CIMMYT), Addis Ababa, Ethiopia

**Keywords:** Climate change, Hydrology

## Abstract

High-resolution climate model projections for a range of emission scenarios are needed for designing regional and local adaptation strategies and planning in the context of climate change. To this end, the future climate simulations of global circulation models (GCMs) are the main sources of critical information. However, these simulations are not only coarse in resolution but also associated with biases and high uncertainty. To make the simulations useful for impact modeling at regional and local level, we utilized the *bias correction constructed analogues with quantile mapping reordering* (BCCAQ) statistical downscaling technique to produce a 10 km spatial resolution climate change projections database based on 16 CMIP6 GCMs under three emission scenarios (SSP2-4.5, SSP3-7.0, and SSP5-8.5). The downscaling strategy was evaluated using a *perfect sibling* approach and detailed results are presented by taking two contrasting (the worst and best performing models) GCMs as a showcase. The evaluation results demonstrate that the downscaling approach substantially reduced model biases and generated higher resolution daily data compared to the original GCM outputs.

## Background & Summary

The unavailability of high-resolution climate data is an important obstacle for local climate change impact studies on agriculture and biodiversity. The analysis of climate change impact requires forcing data at sufficiently high-resolution for crop and hydrological models, both temporally (daily) and spatially (~1–10 km). Climate projection data produced by GCMs only exist at a coarse spatial resolution (typically a horizontal grid spacing >70 km) and with a high spread between models^1^. This variability (i.e., the range between models) induces uncertainties that propagate to calculated impacts (e.g., model outputs), which makes the studies less relevant to decision-making^2^. In the recent decade, this high uncertainty has been addressed using multi-model ensemble approaches which heavily lean on the use of multiple GCMs in crop modeling^3–6^ and hydrological modeling^7–9^. These studies require multiple state variables, in general from different emission scenarios, to be bias-corrected and downscaled to a higher temporal and spatial resolution than the original GCMs^10–13^. Downscaling is mainly necessary since GCMs do not resolve small-scale climate-affecting land surface features such as smaller mountain ranges. If GCM outputs are not appropriately corrected and downscaled, the process leads to highly erroneous results^14^.

For a country like Ethiopia with very diverse climate regimes modulated by its complex topography^15,16^, local planning, and monitoring are unimaginable without high-resolution climate data. In this regard, downscaling bridges the mismatch between coarse resolution climate outputs and data requirements by impact models^17,18^. Efforts have been made globally and regionally, including by WorldClim, CCAFS, and ref. ^19^, to create high-resolution climate projection datasets encompassing Ethiopia. For example, WorldClim (https://www.worldclim.org/data/cmip6/cmip6climate.html) has publicly released high-resolution CMIP6 projection datasets for 23 GCMs and four Shared Socio-economic Pathways (SSPs) at resolutions of 10 minutes, 5 minutes, 2.5 minutes, and 30 seconds. However, these datasets are limited in that they are only available as monthly values averaged over 20-year periods, which may not meet the demands of ecological impact models that require daily data. Additionally, the datasets are produced to provide a global quick overview of the projected climate, and the methods (https://worldclim.org/data/downscaling.html) used ignored the temporal trends and extremes and hence limiting its application in impact assessment studies. Similarly, CCAFS datasets (http://www.ccafs-climate.org), which use a simple bias correction technique called the Delta method, also have a limited temporal resolution, with only 30-year averages available. Ref. ^19^ have produced similar datasets for regions of East Africa (including Ethiopia), but their datasets are based on station data from 211 stations, and the models they used were limited to two GCMs. Despite these limitations, these datasets can still provide valuable insights into climate trends and support impact assessment studies, but alternative approaches may be necessary to obtain more comprehensive and robust data for specific research needs.

A variety of techniques and approaches exist to downscale coarse resolution GCMs output to a higher resolution. The most frequently used approaches in hydrological and agricultural studies are *statistical* and *dynamical downscaling* methods. The one difference between the two approaches lies in the required computational resources, especially when daily data are to be produced, but also in the accuracy of the results^20^. Regional Climate Models (RCMs) are used to dynamically downscale GCM outputs to a finer resolution by using the boundary and initial conditions from a GCM as input^21^. However, dynamical downscaling through RCMs is computationally very expensive^21^. This explains why high-resolution data from sets of GCMs under different emission scenarios are rarely produced using RCMs^22,23^.

Statistical downscaling is a technique used to generate high-resolution climate data by relating large-scale climate variables, such as temperature and precipitation, to local-scale variables^2,24,25^. Bias correction (BC) statistical downscaling (SD) is a popular technique used to produce high-resolution climate data from General Circulation Model (GCM) outputs, and they are often computationally efficient compared to other methods. The approach involves first applying a statistical bias correction to the GCM outputs using observations at the GCM grid and then using spatial downscaling to generate a more fine-resolution result^14,26^. Bias correction is a statistical technique used to adjust data that has a bias or systematic error so that it reflects the true values. The bias correction process can be accomplished through several methods, such as applying a simple change factor (i.e., ‘delta’) between the GCM output and observation^2,27^, or by using quantile-mapping techniques to match the GCM output to observations^28–31^. Once the bias correction is complete, the downscaled data can be generated using spatial downscaling techniques^14^.

The objective of this study was to produce a database of high-resolution bias-corrected climate change projections for Ethiopia. To this end, we bias-corrected daily maximum and minimum temperatures from 16 GCMs and daily precipitation from 12 GCMs and downscaled it to a 10 km spatial grid covering all of Ethiopia using the bias correction constructed analogues with quantile mapping reordering (BCCAQ) statistical downscaling technique. GCM outputs under three emission scenarios, also called Shared Socioeconomic Pathways (SSPs), were taken from the Coupled Model Intercomparison Project Phase 6 (CMIP6). As reference data, we used 5 km resolution gridded data for the base period (1983–2012). Furthermore, a *perfect sibling* framework (see section 2) was employed to assess the value of the downscaling strategy.

Here, we produced high-resolution daily precipitation and temperature data through a gridded statistical downscaling method called BCCAQ^32^. The method significantly reduced the biases between the GCMs output and the observation data and minimized the errors in the projections. The data will help overcome limitations in climate change impact studies on crop growth^33–36^ and hydrology^37–39^ in Ethiopia resulting from the insufficient number of GCM used. Our evaluation allows a clear perspective on individual GCMs by means of correlations, biases, and temporal evolution. Our study will make a valuable contribution to future model comparison efforts in Ethiopia and beyond by revealing the differences between the various models. In particular, our findings indicate that the simulations produced by the two Norwegian GCMs (NorESM2-LM and NorESM2-MM) may be implausible despite their strong performance in East Africa^40^. Conversely, the consistent results observed across Ethiopia’s diverse regions suggest that for the purposes of our study, fewer homogeneous regions, similar to the approach taken by ref. ^41^ may be sufficient. Finally, the dataset will help researchers examine a wide range of scenarios, which can help inform more comprehensive and robust adaptation strategies.

## Methods

### Gridded observation data

Daily climate data is available for the study area (Supplementary Fig. 1a) from CHIRPS (Climate Hazards Group InfraRed Precipitation with Stations) for precipitation^42^ and from CHIRTS (Climate Hazards Group InfraRed Temperature with Stations) for temperature^43^ (Supplementary Fig. 1b–d). The climate hazards group database provides free daily climate data with a gridded 5 km spatial resolution with quasi-global coverage (50S–50 N) (ftp://ftp.chg.ucsb.edu/pub/org/chg/products/ or https://www.chc.ucsb.edu/products). The data were generated in several stages by blending satellite records and *in situ* station data. They are available for precipitation since 1981 and for temperature from 1983 to 2016. The data have been evaluated for their reliability to represent the climatology and major meteorological systems in Ethiopia^44–48^ and elsewhere^49–52^.

The 16 GCMs from CMIP6 used for downscaling are listed in Table 1. The three selected SSPs are SSP2-4.5, SSP3-7.0, and SSP5-8.5 representing the medium, medium-high, and high-forcing scenarios, which in turn were based on so-called middle-of-the-road, regional rivalry, and fossil-fueled socioeconomic development scenarios, respectively^53,54^. The data of precipitation, maximum and minimum temperature, at a horizontal grid spacing ranging from about 70 km to 400 km were accessed from the CMIP6 web data portal^55^ (https://esgf-node.llnl.gov/search/cmip6). To be consistent across the models, all downloaded GCM data belonged to the ‘r1i1p1f1’ variant (standing for 1^*st*^ realization, 1^*st*^ initialization, 1^*st*^ physics, and 1^*st*^ forcing). A 30-year period (1983–2012) was adopted as a baseline period for method validation and a period centered at the 2050 s (2036–2065) was adopted for the projected climate for the case studies.

**Table 1:** List of GCMs used in the study and availability of data with respect to maximum (Tmax) and minimum (Tmin) temperatures and precipitation (Pr) as well as spatial resolution.

Model name	Institution name	Tmax	Tmin	Pr	Resolution (lon. by lat.)
ACCESS-CM2^87–90^	Commonwealth Scientific and Industrial Research Organization (CSIRO) and Bureau of Meteorology (BOM), Australia	✓	✓	✓	1.9° × 1.3°
ACCESS-ESM1-5^91–94^	Commonwealth Scientific and Industrial Research Organization (CSIRO) and Bureau of Meteorology (BOM), Australia	✓	✓	✓	1.9° × 1.3°
AWI-CM-1-1-MR^95–98^	Alfred Wegener Institute, Helmholtz Centre for Polar and Marine Research, Am Handelshafen 12, 27570 Bremerhaven, Germany	✓	✓	*o*	0.9° × 0.9°
CMCC-CM2-SR5^99–102^	Fondazione Centro Euro-Mediterraneo sui Cambiamenti Climatici, Lecce 73100, Italy	✓	✓	✓	1.3° × 0.9°
EC-Earth3-Veg^103–106^	EC-Earth-Consortium	✓	✓	*o*	0.7° × 0.7°
EC-Earth3^107–110^	EC-Earth-Consortium	✓	✓	*o*	0.7° × 0.7°
GFDL-ESM4^111–114^	NOAA Geophysical Fluid Dynamics Laboratory	✓	✓	✓	1.3° × 1.0°
INM-CM4-8^115–118^	Institute for Numerical Mathematics	✓	✓	✓	2.0° × 1.5°
INM-CM5-0^119–122^	Institute for Numerical Mathematics	✓	✓	✓	2.0° × 1.5°
IPSL-CM6A-LR^123–126^	Institut Pierre Simon Laplace, Paris 75252, France	✓	✓	*o*	2.5° × 1.3°
MIROC6^127–130^	Japan Agency for Marine-Earth Science and Technology, Kanagawa 236-0001, Japan	✓	✓	✓	1.4° × 1.4°
MPI-ESM1-2-HR^131–134^	Max Planck Institute for Meteorology, Hamburg 20146, Germany	✓	✓	✓	0.9° × 0.9°
MPI-ESM1-2-LR^135–138^	Max Planck Institute for Meteorology, Hamburg 20146, Germany	✓	✓	✓	1.9° × 1.9°
MRI-ESM2-0^139–142^	Meteorological Research Institute	✓	✓	✓	1.1° × 1.1°
NorESM2-LM^143–146^	NorESM Climate modeling Consortium, Norway	✓	✓	✓	2.5° × 1.9°
NorESM2-MM^147–150^	NorESM Climate modeling Consortium, Norway	✓	✓	✓	1.3° × 0.9°

### Delineating homogeneous precipitation zones

**Fig. 1:**
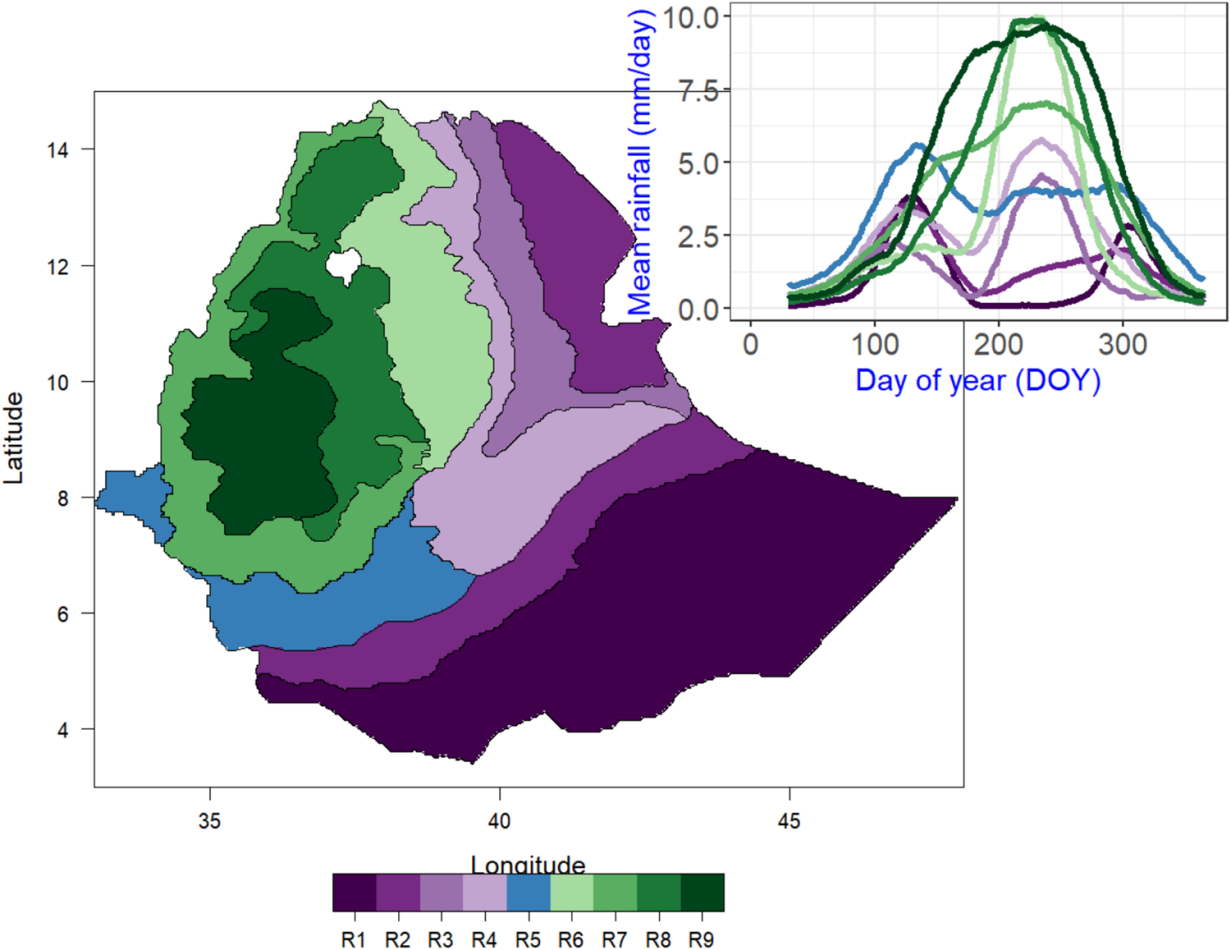
Homogeneous precipitation clusters (sub-regions) based on climatological (1983-2012) mean daily precipitation.

Ethiopia is characterized by diverse climate regions modulated by complex topography (Supplementary Fig. 1a) where precipitation is a crucial component, particularly for the agricultural sector^15,16^. To acknowledge this heterogeneity and better describe the follow-up analysis, we map out the long-term mean daily precipitation into homogeneous precipitation clusters using the CLARA algorithm (Clustering for LARge Application)^56^. In CLARA, a sample is drawn from a large dataset and partitioned into k clusters based on the K-medoid approach. The remaining data are then assigned to the nearest k clusters. The clustering resulted in nine homogeneous precipitation regions (R1-R9) with distinct seasonal patterns (Fig. 1), which are roughly like ecozones in previous studies^57–59^. Most regions receive precipitation mainly in June-September (months JJAS) except R1 and R2 where the main season is March-May (MAM) with a short rain in October. These relatively homogeneous (i.e., in terms of annual precipitation cycles) sub regions were used to facilitate aggregated comparisons between the different outputs before and after downscaling.

#### The downscalinsg approach

**Fig. 2:**
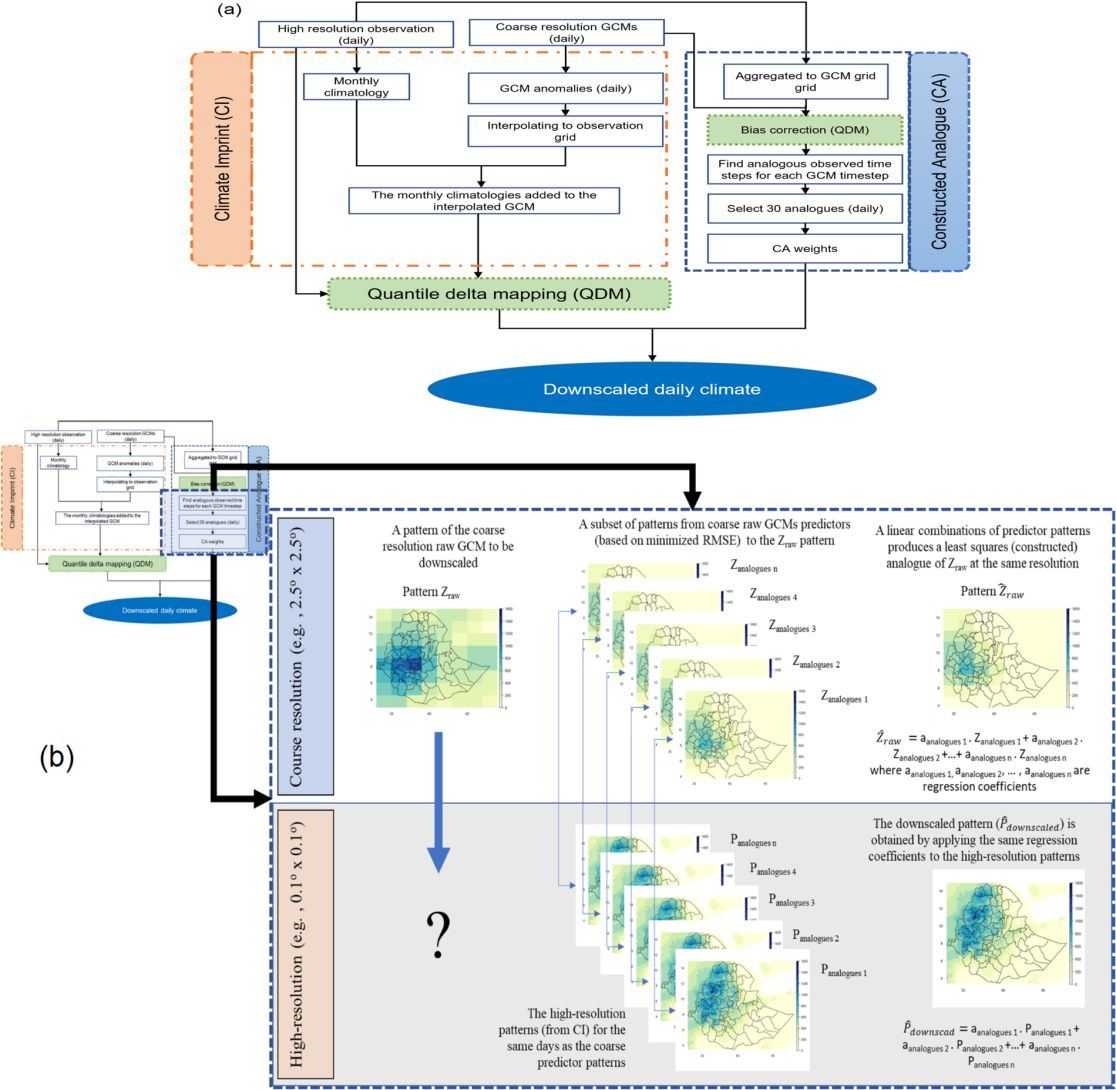
Schematic flow diagram of (**a**) the BCCAQ downscaling technique showing the steps and processes followed in the study and (**b**) the method of constructed analogues (CA) modified from ref. ^62^.

In this study, we followed an approach called *bias correction constructed analogues with quantile mapping reordering (BCCAQ)* to downscale the coarser scale GCM outputs to a finer scale^26,32,60,61^. Compared to other statistical downscaling approaches, BCCAQ has shown to reach a better skill in representing extremes^32,60,61^. In the BCCAQ approach (Fig. 2a), two stage independent calculations, Climate Imprint (CI) and Constructed Analogue (CA)^26,62^ are calculated in parallel, which we describe in more detail below.

**Step A (Climate Imprint, CI**, **Fig.** 2a**):** ***Step A1:-*** Calculating monthly climatologies separately from the daily observation and raw historical GCM datasets for the reference period (1983–2012) for the variable of interest (e.g., temperature) as follows (**i-v**).

i. Extract the data for the month of interest (e.g., January) for each year in the reference period.
ii. Calculate the average of all the daily values for each year.
iii. Repeat step **ii** for all the years in the reference period.
iv. Calculate the average of all the yearly averages. This gives the monthly climatology for the variable of interest for the month of January.
v. Repeat these steps for all the months in the reference period.

***Step A2:-*** Prepare raw GCM daily anomalies (1983–2100): For each day of the year, subtract the corresponding monthly climatology value for that day from raw GCM daily values for that day. This will give the daily anomaly for each day of the year.

***Step A3:-*** Interpolating the raw GCM daily anomalies (1983–2100) to the same spatial resolution as the observation data (fine resolution): This will result in finer resolution data with the same temporal length as the coarser-resolution GCM outputs.

***Step A4:-*** Apply the monthly climatology of observation to the interpolated raw GCM daily anomalies (1983–2100). For each day of the year and for each grid cell, add the corresponding monthly climatology value of the observation for that month to the interpolated raw GCM daily anomaly value.

***Step A5:-*** Bias-correcting the raw GCM from ***Step A4*** with the observation using quantile delta mapping (QDM, see below for detail description) and the data then will be ready for the next stage of the downscaling process.

**Step B (Constructed Analogue, CA, Illustrated in** **Fig.** 2b**):**

***Step B1:-*** Aggregating the finer scale daily observation data to the GCMs grid.

***Step B2:-*** Bias-correcting the raw GCM with the observation using QDM (see below for detailed description).

***Step B3:-*** Searching for a subset of analogues GCM: For a given day of the year in the bias corrected GCM weather pattern to be downscaled (i.e., target pattern), a search is conducted for observed steps (i.e., +/− 45 days of form that particular day) to find a subset of analogues GCM weather patterns with the target pattern. Here, the top 30 closest time steps (days) that have the closest similarity (analogues) to a given GCM daily weather pattern are selected based on minimum root mean square error (RMSE)^62^.

***Step B4:-*** The CA weights (regression coefficients) are then determined via ridge regression of the CA time steps (i.e., between the target weather pattern and the selected top 30 closest fields)^26^.

***Step B5:-*** Linear combinations: For the given timestep, these derived CA weights (regression coefficients) in Step B4 are then used to linearly combine the 30 corresponding bias-corrected CI outputs in ***Step A3*** to create the spatially downscaled high-resolution data.

***Step B6:-*** Reordering: The daily downscaled data are reordered within a given month based on the daily bias-corrected CA ranks. The reordering process ensures a better representation of event-scale spatial variability and a broadly consistent long-term trend between the downscaled outputs and bias-corrected CI^32,61,63^ (***Step A3***). Further details can be found in ref. ^26,32,60,62^.

#### Quantile delta mapping (QDM)

For a given meteorological state variable of interest, QDM^28–31^ starts by estimating the empirical cumulative distribution functions (CDFs) of the raw historical GCM series (*F*_*h*_) and that of the observations data (*F*_*o*_) during a reference period (1983–2012). Concurrently, it prepares the CDF of the raw GCM projected series (*F*_*m*_ (*t*)), which is estimated over a time *t*, using 30-year moving windows. The “30-year sliding window” here refers to a moving timeseries of 30 years. For instance, to perform QDM for the projection years (*t*) 2035, 2036, 2037, etc., timeseries of 30 years i.e., 2021–2049 (centred at 2035), 2022–2050 (centred at 2036), 2023–2051 (centred at 2037), etc., will be taken. Then, the non exceedance probability, *ϵ*(*t*) associated with the raw GCM projected value at time *t* is calculated within the projection period based on estimated CDF, *F*_*m*_ (*t*). Next, the inverse CDFs of the raw historical GCM series ($\left({F}_{h}^{-1}\left[\epsilon \left(t\right)\right]\right)$) and observations $\left({F}_{o}^{-1}\left[\epsilon \left(t\right)\right]\right)$ during a reference period (1983–2012), and that of the raw GCM projected series $\left({F}_{m}^{-1}\left[\epsilon \left(t\right)\right]\right)$ are all evaluated at the nonexceedance probability (*ϵ*(*t*)) associated with the modelled value at time *t*. Then, for precipitation (temperature), the relative (absolute) change in quantiles between the reference periods and project time *t*, Δ*t* can be calculated as the ratio (difference) of the $\left({F}_{m}^{-1}\left[\epsilon \left(t\right)\right]\right)$ and $\left({F}_{h}^{-1}\left[\epsilon \left(t\right)\right]\right)$. Finally, the bias-correction results *δ*(*t*) in the future period at time *t* equals ${F}_{o}^{-1}\left[\epsilon \left(t\right)\right]$ × Δ*t* for precipitation, and ${F}_{o}^{-1}\left[\epsilon \left(t\right)\right]$ + Δ*t* for temperature.

#### Evaluation approach

For computational reasons, the observation data (daily minimum and maximum temperature, and precipitation) were first aggregated from 5 km to 10 km resolution using bilinear interpolation. Bilinear interpolation is a widely used technique in climate studies to re-grid data e.g.^64–66^. The final downscaled GCM outputs were produced for all three emission scenarios until 2100 (SSP2-4.5, SSP3-7.0, and SSP5-8.5).To evaluate the impact of the chosen downscaling strategy we used an approach called the *perfect sibling* (PS) framework^2,27,67^. This approach serves to evaluate the evolution of future simulation when no observations exist such as in the case of future climate. In the *perfect sibling* framework, a random GCM will be selected from the list of available GCMs as pseudo-observations for a certain reference period. The rest of the GCMs are used as a prediction of the pseudo-observations and, hence, comparisons can be made between the performance of the individual GCMs for the selected reference period. In this study, MPI-ESM1-2-HR GCM was selected as a reference simulation (mimicking observations) or *perfect sibling*. The remaining GCMs were compared to it for the base period (1983–2012) and the 2050 s periods (2036–2065). A two-stage evaluation was conducted i) for the base period and ii) for the projected climate. First, Pearson correlation coefficients (r) and root mean squared errors (RMSE) were calculated between the simulated and gridded observations of annual mean maximum and minimum temperature (JJAS total precipitation) for the base period (1983–2012) for each grid cell. This allowed us to compare the skill of the models in representing the base climate before and after the application for the downscaling. Next, for the future projection period, the RMSEs between the *perfect sibling* and the rest of the GCMs were calculated to assess the skill of the GCMs in reproducing the *perfect sibling* for the whole grid cells. Here, the primary objective of the evaluations was not to compare the performance of different models but rather to assess the impact of the downscaling strategy. However, in order to provide a more comprehensive analysis of the evaluation’s temporal and spatial aspects, we selected two contrasting Global Climate Models (GCMs): CMCC-CM2-SR5, which had relatively poor performance, and MPI-ESM1-2-HR, which had better performance in reproducing observations during the reference period (prior to downscaling). To demonstrate the differences before and after the application of the downscaling process, we compared the cumulative density functions (CDFs) of various datasets, including observations, historical raw GCMs data, future raw GCMs data, and downscaled data. The term “bias” in this study refers to the absolute difference between the values of two fields, specifically the modeled value minus the observed value. The downscaling was performed using the High-Performance Computing Cluster bwUniCluster 2.0 (https://wiki.bwhpc.de/e/Main_Page). All statistical and spatial analyses in this study were performed using R, an open-source statistical software^68^. Downscaling was conducted using the ClimDown^69^ package. The ncdf4^70^ and cmsafops^71^ packages were used to read and edit the GCMs’ NetCDF files. Regionalization of the country into homogeneous precipitation zones was done using the cluster package^72^. All plots were produced using packages ggplot2^73^, cowplot^74^, gridExtra^75^, raster^76^, rasterVis^77^ and rgdal^78^ packages.

## Data Records

The database of the statistically downscaled daily precipitation (pr), maximum temperature (tasmax) and minimum temperature (tasmin) for the current climate (1975–2015) and future scenarios (2016–2100) under the three SSPs (SSP2-4.5, SSp3-7.0 and SSP5-8.5) are stored in the CIMMYT repository^79^. The total file size is ~400 GB where each file is stored as a self-describing NetCDF file format with the same naming as their original GCM names with ‘Eth_’ as a prefix. For instance, the file name “Eth_pr_ACCESS-CM2_ssp370.nc” refers to the downscaled daily precipitation (pr) data for the GCM (ACCESS-CM2) under the SSP3-7.0 (ssp370) emission scenario and “Eth_tasmax_EC-Earth3-Veg_ssp245.nc” refers to the downscaled daily maximum temperature (tasmax) data for the GCM (EC-Earth3-Veg) under the SSP2-4.5 (ssp245) emission scenario. The gridded observation data sets are provided by the Climate Hazards Group and are publicly available at https://www.chc.ucsb.edu/data. The original CMIP6 data were downloaded from the Earth System Grid Federation (ESGF) website (https://esgf-node.llnl.gov/search/cmip6/), with detailed information about the data, including the terms of use.

## Technical Validation

For the purposes of the demonstrations, we show the comparisons for the SSP3-7.0 emission scenario for the mid of the century (2036–2065) centered on the 2050 s for precipitation and temperature. We also show the comparison of the CDFs for the annual mean maximum and minimum temperature and JJAS total precipitation under each homogeneous precipitation region. The respective spatial plot for the long-term mean values where the CDFs are calculated from are also presented for the two contrasting GCMs.

### Precipitation

**Fig. 3:**
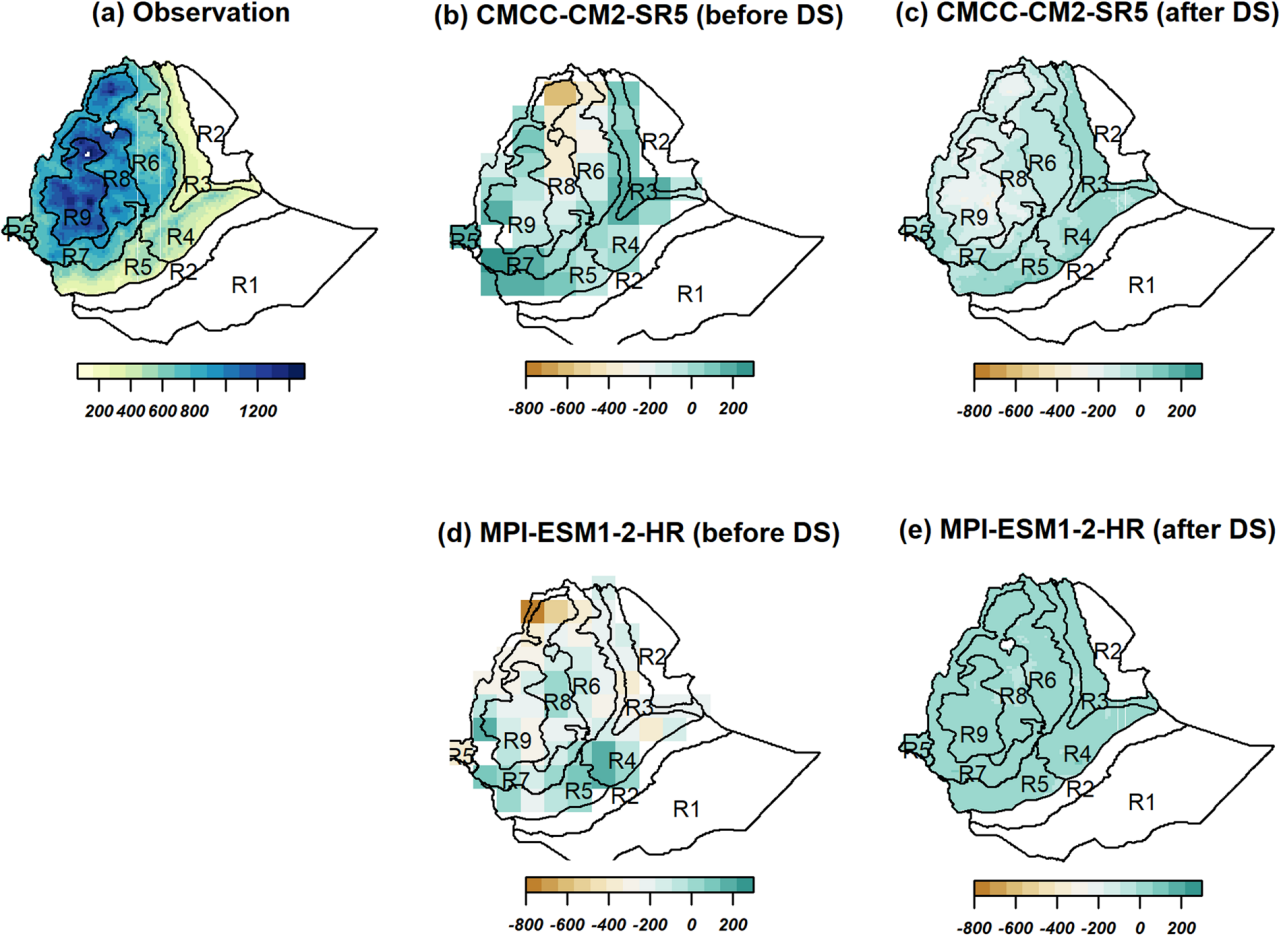
Spatial plots comparing averaged observation (1983-2012) JJAS total precipitation (**a**) and the absolute biases for the two models, CMCC-CM2-SR5 (**b**,**c**) and MPI-ESM1-2-HR (**d**,**e**) before and after the downscaling (units in mm). The base map shows the nine homogeneous precipitation subregions in text (R1, R2, …, R9).

**Fig. 4:**
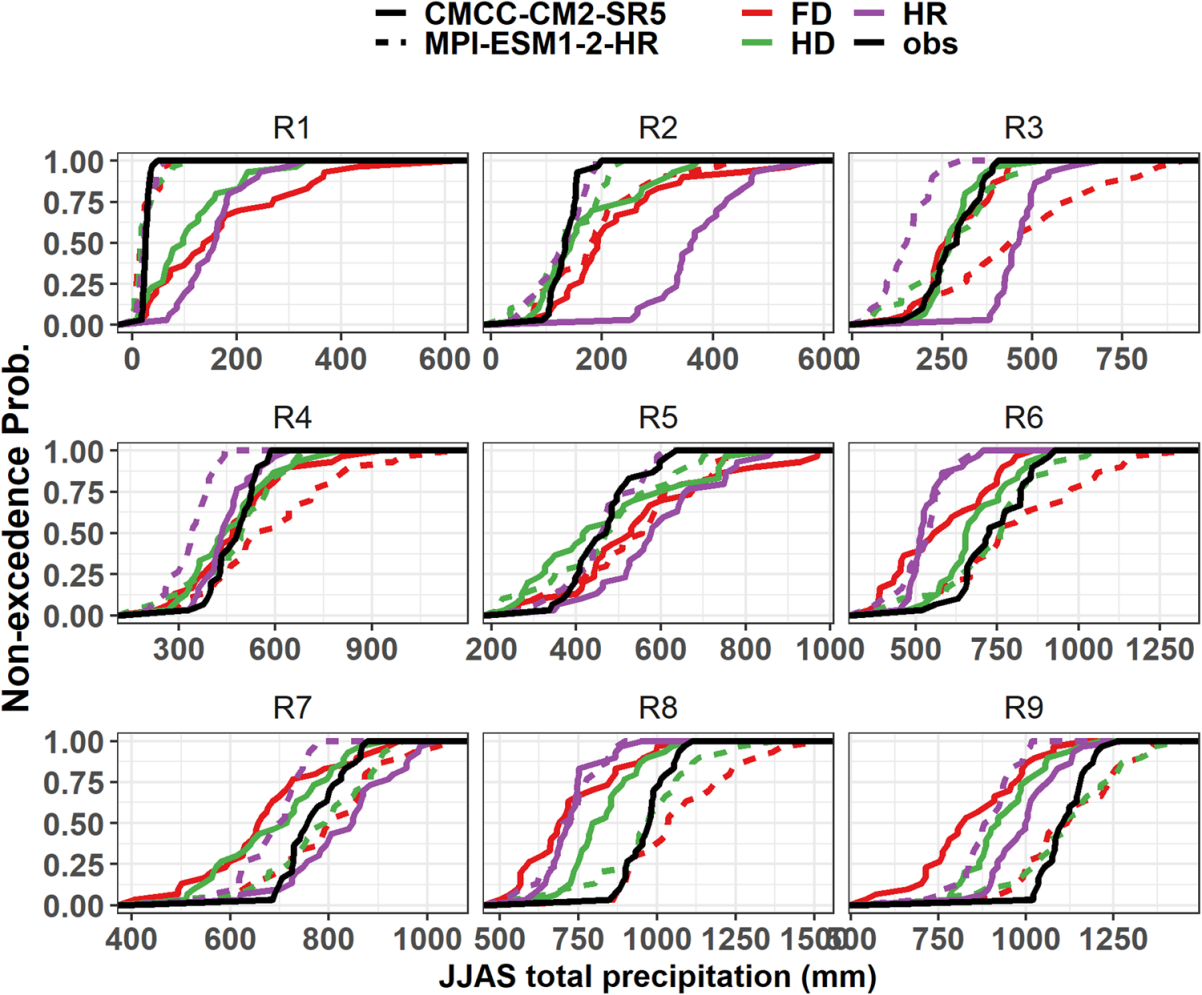
Cumulative density function plots for JJAS total precipitation for the nine homogeneous precipitation subregions (R1, R2, …, R9) under SSP3-7.0 scenario. HR represents models’ historical climate before downscaling and, HD and FD represent models’ historical climate and future projections after downscaling. Obs represent the observation data for the base period.

Table 2 presents the 5th percentile, the median, and the 95th percentile of the spatial distribution model biases aggregated across the country of the 30-year JJAS (June-July-August-September) total precipitation for the two selected contrasting GCMs (the least performed CMCC-CM2-SR5 and most performed, MPI-ESM1-2-HR) and the corresponding observations (see Supplementary Table 1 for model evaluation results for the rest of the models). Spatially aggregated values of the GCMs and the respective biases (defined as the difference between model and observation, defined by bias = modeled value – observed value) with the observation before and after the downscaling process were compared. Table 2 also presents the projected values for the 2050 s under the SSP3-7.0 emission scenario with the respective biases and relative percentage changes after the downscaling. The ranges in the remainder of this section always refer to the 5th and 95th quantile ranges, unless stated otherwise. The JJAS period is the main rainy season for the large parts of the country except for eastern and southeastern parts^57–59^, where the long term mean JJAS total precipitation ranges from 250 to 1200 mm (Table 2 and Fig. 3a). Figure 3 shows the spatial plots of the two GCMs before and after downscaling with the corresponding biases during the base period and mean JJAS total precipitation. The subplots in the figure show marked spatial differences between the two GCMs both before and after downscaling. Before downscaling, both models show largely wet biases with the JJAS climatology values ranging from 345–1100 mm for the CMCC-CM2-SR5 (i.e., least performed) and 80–1010 mm for the MPI-ESM1-2-HR (i.e., most performed) (Table 2). Both models also show dry biases up to 600 mm over the northern highland parts of the country. The evaluation of CMIP5 models for Ethiopia also shows a dry bias over the highlands, underrepresenting orographic uplift^80^. Whereas the corresponding countrywide area-averaged JJAS climatology values range from 260 to 1000 mm and 260 to 1200 mm by the CMCC-CM2-SR5 (i.e., least performed) and MPI-ESM1-2-HR (i.e., most performed), respectively, after the application of the downscaling. The respective biases range from −360 to 205 mm and from −370 to 145 mm before downscaling and the biases decreased to a range of −195 to 35 mm and −15 to 30 mm after downscaling for the CMCC-CM2-SR5 (i.e., least performed) and MPI-ESM1-2-HR (i.e., most performed), respectively, showing an overall improvement due to the downscaling. However, the CDF plots in Fig. 4 highlight the importance of visually inspecting the spatial variations of the zonal averages across the homogeneous precipitation regions. It is very clear that, before downscaling, the CMCC-CM2-SR5 (i.e., least performed) outputs could not reproduce the CDFs of the observations well in many the regions (Fig. 4). Figures 3, 4 also show that the MPI-ESM1-2-HR (i.e., most performed) model’s relatively better representation is only valid on a country scale. However, the improvement due to the downscaling still holds to some extent in each homogeneous zone.

**Table 2:** Summary statistics of the countrywide area-averaged quantiles of mean bias, RMSE, and Pearson correlation coefficient (r) for JJAS total precipitation for the two selected contrasting GCMs (the least performed CMCC-CM2-SR5 and most performed, MPI-ESM1-2-HR) and the corresponding projected relative percentage changes by the 2050 s compared with the observation data (1983–2012).

Historic evaluation	Models	Bias (mm)			RMSE	r
		q5	q50	q95		
Before downscaling	CMCC-CM2-SR5	−360	3	205	76.2	0.46
	MPI-ESM1-2-HR	−369	−179	144	56.1	0.60
Downscaled	CMCC-CM2-SR5	−194	−47	35	67.1	0.33
	MPI-ESM1-2-HR	−13	2	30	55.3	0.65
Projected changes based on the downscaled data		**(%)**				
	CMCC-CM2-SR5	−30	−17	40		
	MPI-ESM1-2-HR	−4	10	67

Based on the downscaled data, the projected JJAS total precipitation quantiles range from 280 to 900 mm and from 350 to 1235 mm for the CMCC-CM2-SR5 (i.e., least performed) and MPI-ESM1-2-HR (i.e., most performed), respectively, for the 2050 s under the SSP3-7.0 emission scenario (Supplementary Fig. 2). The corresponding percentage of change ranges −30 to 40% and from −5 to 65% for the CMCC-CM2-SR5 (i.e., least performed) and MPI-ESM1-2-HR (i.e., most performed), respectively, where mostly a negative (positive) anomalous JJAS total precipitation (up to 500 mm) is projected by the CMCC-CM2-SR5 (MPI-ESM1-2-HR) in large parts of the country (Table 2). This is clearly shown in the CDF plots where large leftward (rightward) shifts are observed particularly for high precipitation receiving regions (i.e., R6 - R8) in the CMCC-CM2-SR5 (MPI-ESM1-2-HR) projected JJAS precipitation. As in the MPI-ESM1-2-HR (i.e., most performed) model, positive projected changes in JJAS total precipitation were also reported for Ethiopia^80^ and across East Africa^81,82^. Figure 7. depicts the spatial pattern of the correlation (Pearson r) and the RMSEs between the observation and the 12 GCM outputs for JJAS total precipitation in the base period (1983–2012) before and after downscaling. The spatial correlation plots reveal that the two models from the Norwegian climate modeling consortium, (i.e., NorESM2-LM and NorESM2-MM), were exceptionally different from the rest of the models with negative correlation with the observation and higher biases (RMSE > 150 mm) for the largest part of JJAS precipitation benefiting regions of the country (Fig. 7a,b). Despite positive spatial correlations, higher biases were also observed in MIROC6 and ACCESS-ESM1-5 models for a considerable part of the country. The application of the downscaling strategy reduced the biases (RMSE < 150 mm) across a large part of the country in all the GCMs except for those two GCMs (Fig. 7c,d). Despite downscaling, the two Norwegian climate models (NorESM2-LM and NorESM2-MM) still exhibit negative correlations and higher biases errors. Moreover, the downscaling process led to an increase in both the intensity and spatial extent of the root mean square error (RMSE). Ref. ^83^ also identified those two models as outliers compared to other models. Figure 8 illustrates a comparison between the RMSE errors in the projected JJAS (June-July-August-September) total precipitation for the 2050 s under the SSP3-7.0 emission scenario, before and after applying downscaling techniques. The comparison is made between a *perfect sibling* projection and the other GCMs during the same projected climate period. The evaluation with the *perfect sibling* framework (Fig. 8 & Supplementary Table 2) shows that the downscaling substantially minimized the bias in the projected climate outputs (the mean RMSE over the whole region denoted by E measured in mm/season are shown on the top of each figure) for all models except for the two Norwegian models. The reduction in the errors ranges from 125 mm/season (~40% lower) for ACCESS-CM2 to 340 mm/season (~80% lower) for MIROC6.

### Temperature

**Fig. 5:**
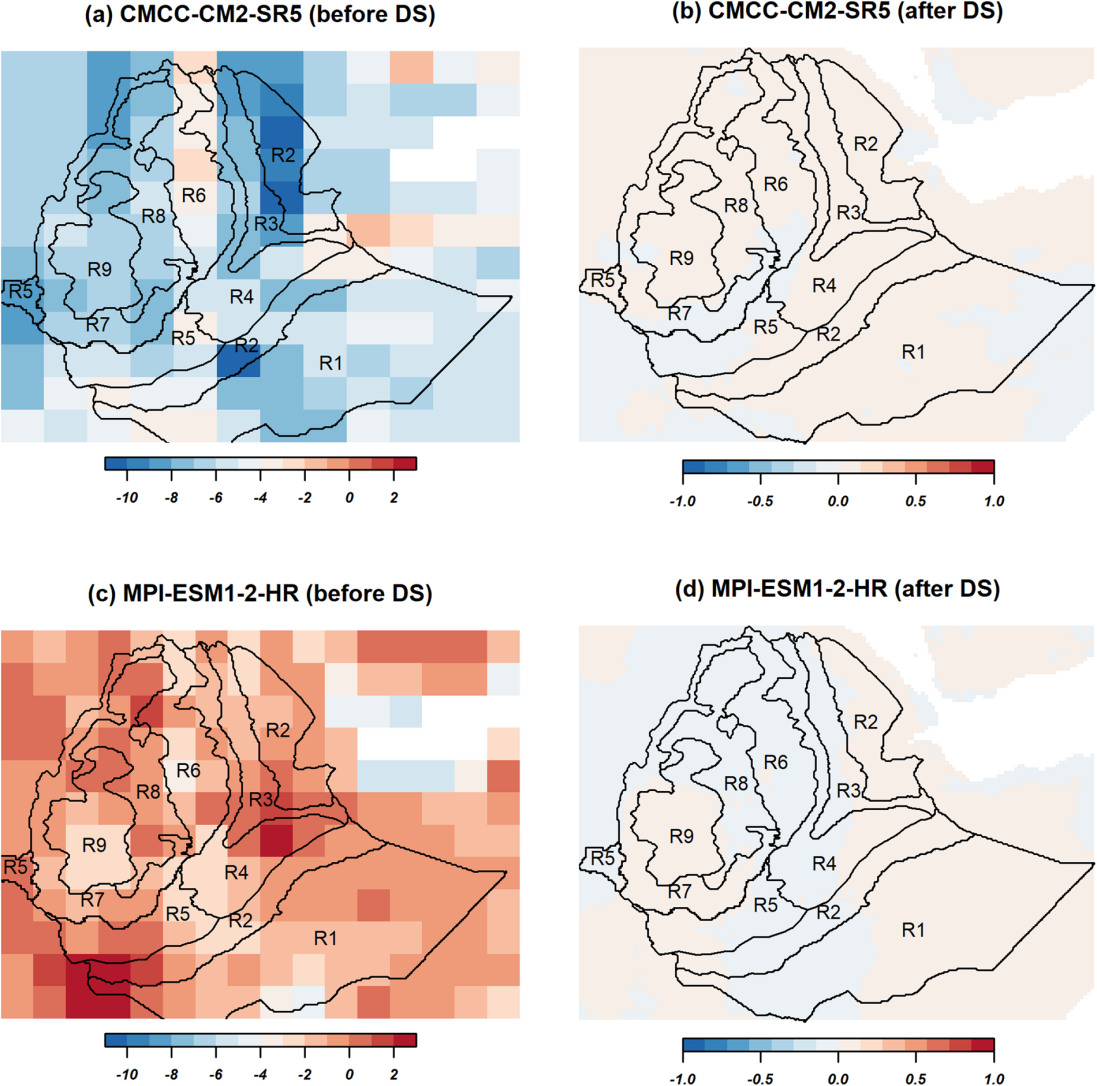
Spatial plots comparing historical (1983-2012) biases in mean annual maximum temperature (°C) before (**a**,**c**) and after (**b**,**d**) downscaling for the two models, CMCC-CM2-SR5 (1st row) and MPI-ESM1-2-HR (2nd row). The base map shows the nine homogeneous precipitation subregions in text (R1, R2, …, R9).

**Fig. 6:**
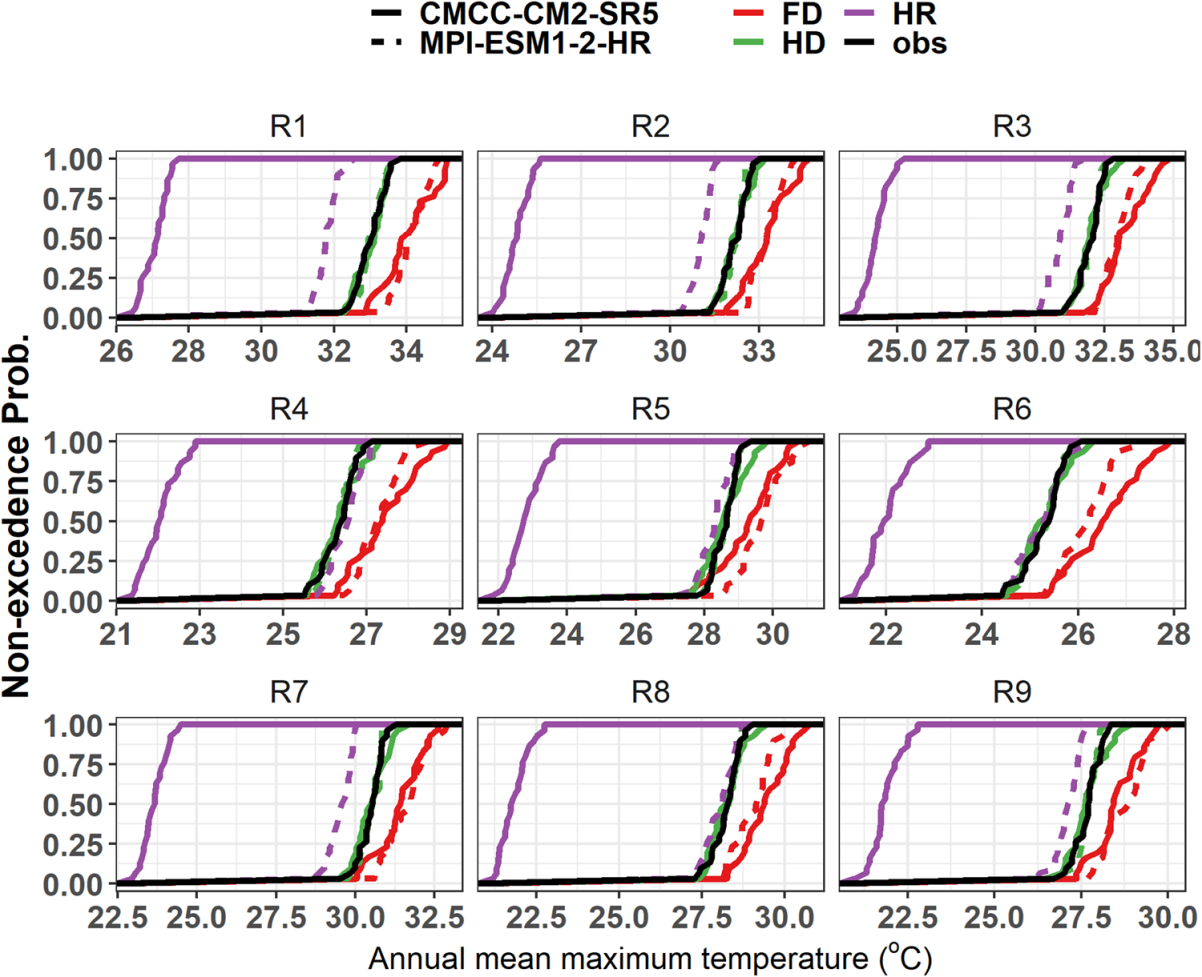
Cumulative density function plots for mean annual maximum temperature (°C) for the nine homogeneous precipitation subregions (R1, R2, …, R9) under SSP3-7.0 scenario. HR represents models’ historical climate before downscaling and, HD and FD represent models’ historical climate and future projections after downscaling. Obs represent the observation data for the base period.

Table 3 presents the 5th percentile, the median, and the 95th percentile of the spatial distribution across the country of the 30-year mean annual maximum and minimum temperature for the two selected contrasting GCMs (the least performed CMCC-CM2-SR5 and most performed, MPI-ESM1-2-HR) and the observation (see Supplementary Tables 2, 3 for model evaluation results for the rest of the models). Aggregated across the country, the observed mean maximum (minimum) temperature ranges from 23 to 37 °C (12 to 27 °C) (Fig. 5a & Supplementary Fig. 4a). For maximum temperature, the historical mean values range from 21 to 30 °C for the CMCC-CM2-SR5 (i.e., least performed) and from 26 to 36 °C for MPI-ESM1-2-HR (i.e., most performed), resulting in absolute biases (*from the observation*) between −8.7 and −3.4 °C and between −3.2 and 1.0 °C, respectively, before downscaling (Fig. 5). Similarly, for minimum temperature, the historical mean values ranged from 21 to 30 °C and from 15 to 27 °C with absolute biases between 1.3 and 8.9 °C and between −2.2 to 2.7 °C for the CMCC-CM2-SR5 (i.e., least performed) and MPI-ESM1-2-HR (i.e., most performed), respectively (Supplementary Fig. 4). The historic GCM outputs of the models were also largely consistence across the country in reproducing the observed CDFs (Fig. 6 & Supplementary Fig. 6). Both models underestimate the maximum temperature in all homogeneous regions except R4-R6 and R8 for MPI-ESM1-2-HR. On the other hand, both models overestimate the minimum temperature in all regions except R2 and R3 for MPI-ESM1-2-HR. By downscaling, the biases of both maximum and minimum temperature were substantial reduced to about 0.1 °C in all regions. After downscaling, the projected (the 2050 s) mean annual maximum (minimum) temperature quantiles range from 25 to 39 °C (from 14 to 29 °C) in both models under the SSP3-7.0 emission scenario. Both models project a mean change of annual maximum (minimum) temperature between 0.6 to 1.4 °C (0.6 to 2.0 °C) (Supplementary Figs. 3a,c, 5a,c). The projected changes of minimum temperature were higher by approximately 0.6 °C in MPI-ESM1-2-HR (i.e., most performed) than in the CMCC-CM2-SR5 (i.e., least performed) (Supplementary Figs. 3b,d, 5b,d).

**Table 3:** Summary statistics of the countrywide area-averaged quantiles of mean bias, RMSE, and Pearson correlation coefficient (r) for annual mean maximum and minimum temperature for the two selected contrasting GCMs (the least performed CMCC-CM2-SR5 and most performed, MPI-ESM1-2-HR) and the corresponding projected relative changes by the 2050 s compared with the observation data (1983–2012).

Variable	Historic evaluation	Models	Bias (°C)			RMSE	r
			q5	q50	q95		
Maximum temperature	Before downscaling	CMCC-CM2-SR5	−8.68	−5.96	−3.44	6.39	0.49
		MPI-ESM1-2-HR	−3.2	−0.75	0.99	2.63	0.70
	Downscaled	CMCC-CM2-SR5	−0.01	0.01	0.02	1.79	0.53
		MPI-ESM1-2-HR	−0.02	0.0	0.01	1.19	0.77
Minimum temperature	Before downscaling	CMCC-CM2-SR5	1.29	4.70	8.86	5.74	0.15
		MPI-ESM1-2-HR	−2.18	0.23	2.67	2.59	0.66
	Downscaled	CMCC-CM2-SR5	0.0	0.01	0.03	1.75	0.49
		MPI-ESM1-2-HR	−0.02	0.0	0.01	1.10	0.75
Maximum temperature	Projected changes by 2050’s based on the downscaled data	CMCC-CM2-SR5	0.6	1.1	1.4		
		MPI-ESM1-2-HR	0.8	1.1	1.3		
Minimum temperature		CMCC-CM2-SR5	0.6	1.1	1.4		
		MPI-ESM1-2-HR	1.4	1.7	2.0

**Fig. 7:**
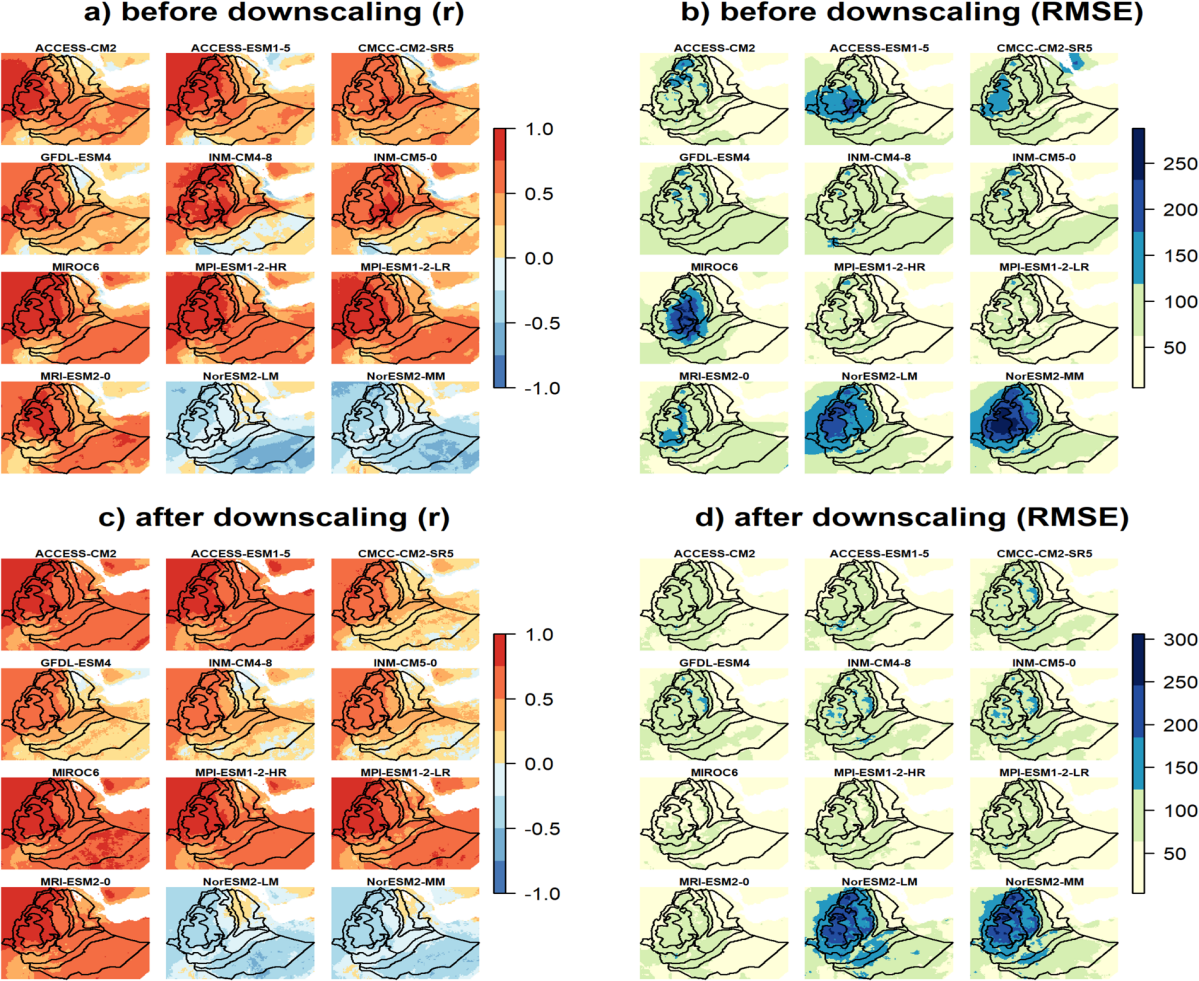
Spatial distribution of Pearson correlation coefficients (left column) and RMSE (right column) between models and observation before (upper row) and after (lower row) downscaling for JJAS precipitation for the period 1983-2012.

**Fig. 8:**
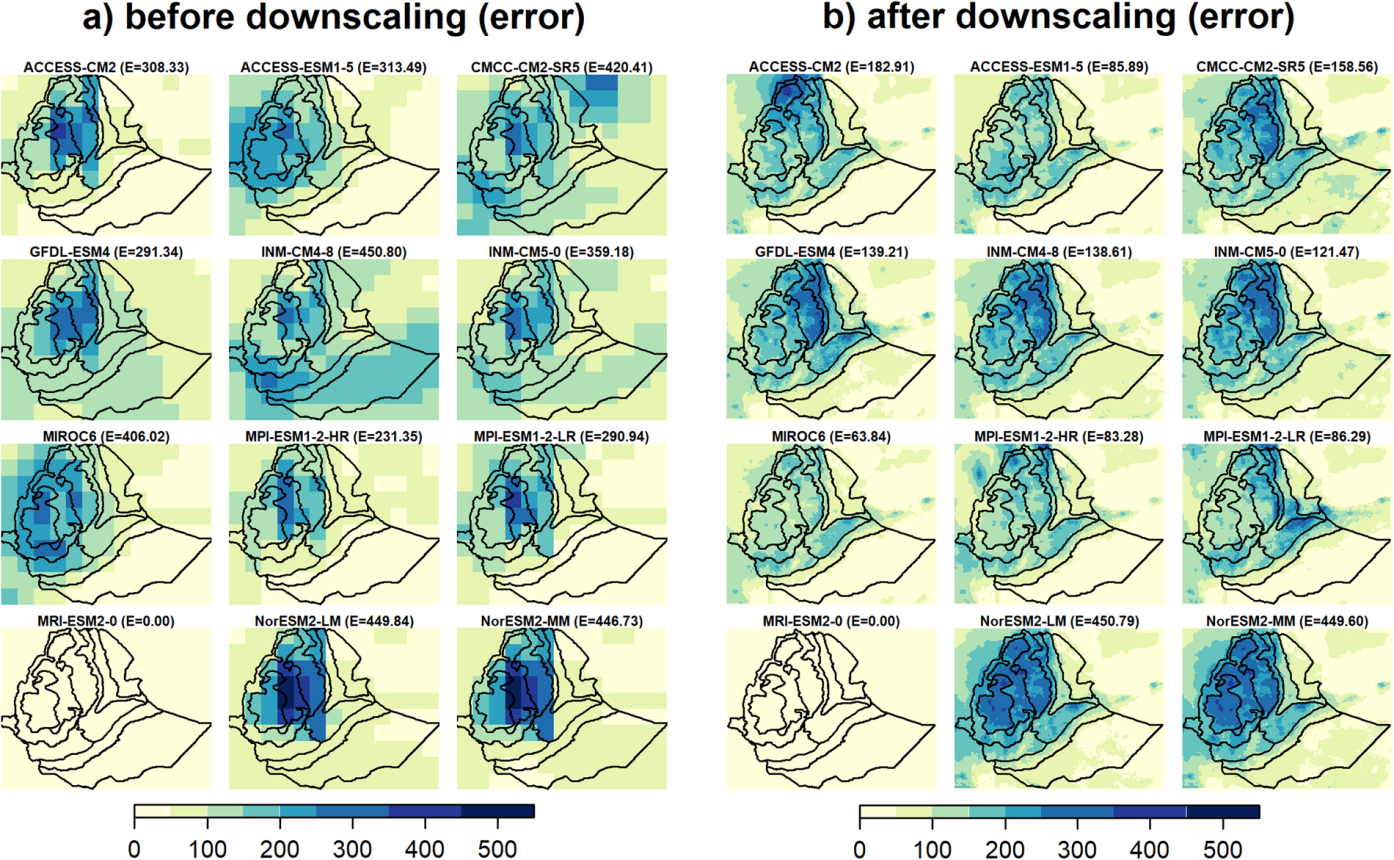
Evaluation (based on RMSE) of GCM outputs using the PS framework (MRI-ESM2-0 selected as a perfect sibling) for JJAS total precipitation (mm) for the 2050s under the SSP3-7.0 scenario, (**a**) before downscaling and (**b**) after downscaling. The mean RMSE errors for the whole region are shown in brackets (E) on top of each plot.

**Fig. 9:**
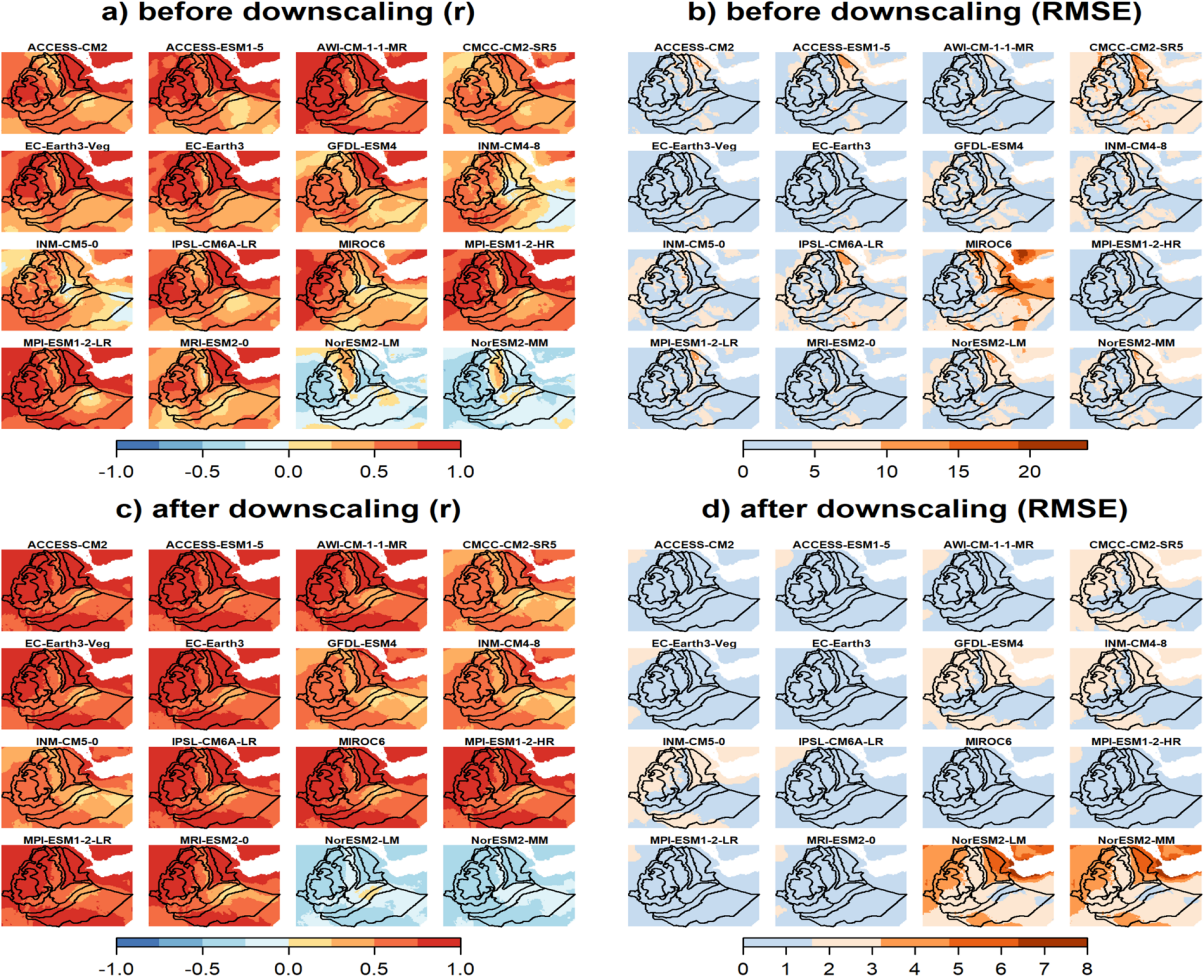
Spatial distribution of Pearson correlation coefficients (left column) and RMSE (right column) between models and observation before (upper row) and after (lower row) downscaling for annual mean maximum temperature for the period 1983-2012.

Figure 9 and Supplementary Fig. 7 illustrate the spatial pattern of Pearson correlation coefficient and of RMSE between the observations and the 16 GCM outputs for maximum and minimum temperature, respectively, in the base period (1983–2012) before and after downscaling. As in precipitation, the correlation plots show that the two Norwegian models were strikingly different from the rest of the models, showing a negative correlation with observations (Fig. 9a,c and Supplementary Fig. 7a,c). For CMCC-CM2-SR5, the figures show also negative correlations across a substantially large part of the country for minimum temperature (Supplementary Fig. 7a). Before downscaling, substantially larger errors (RMSE > 10 °C) in minimum temperature across the peripheral part of the country were produced by all models (Supplementary Fig. 7b). Similarly, particularly in the eastern parts of the country, higher errors in maximum temperature were observed for MIROC6 and CMCC-CM2-SR5 (Fig. 9b). By downscaling, the errors of all models in both maximum and minimum temperatures were substantially decreased to below 2 °C across the country, except the two Norwegian models (Fig. 9d & Supplementary Fig. 7d). In case of CMCC-CM2-SR5, where simulated minimum temperature was mainly negatively correlated with observations, downscaling resulted in a positive correlation across a substantially large part of the country (Fig. 9c & Supplementary Fig. 7c). In case of the two Norwegian models, there was no improvement by downscaling, both in terms of pattern correlation and error minimization. Maximum and minimum temperatures produced by these two models remained negatively correlated with observations and also showed larger errors (RMSE up to 8 °C) in a larger part of the country, particularly in the northwestern and northeastern parts.

**Fig. 10:**
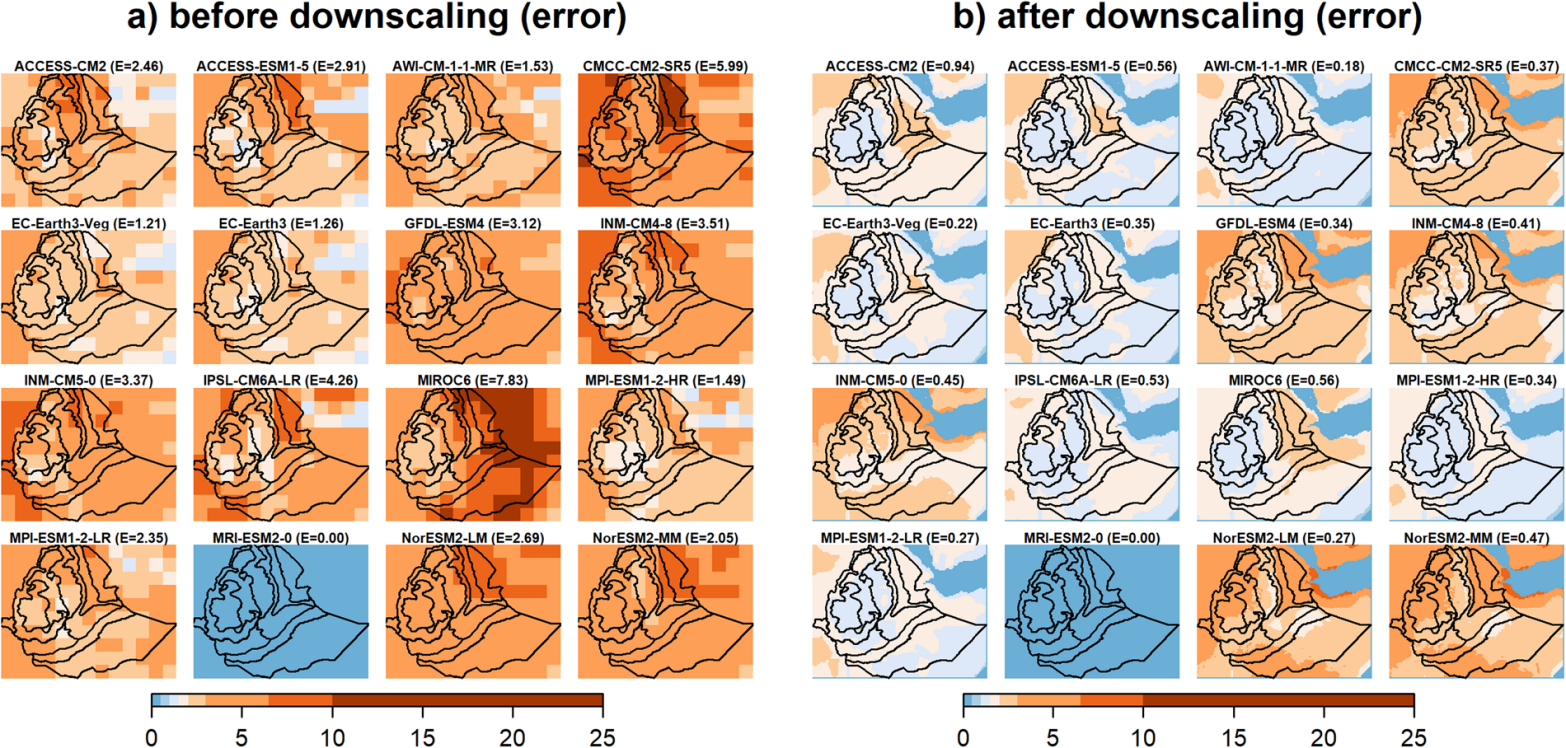
Evaluation (based on RMSE) of GCM outputs using the PS framework (MRI-ESM2-0 selected as a perfect sibling) for annual mean maximum temperature for the 2050s under the SSP3-7.0 scenario, (**a**) before downscaling and (**b**) after downscaling. The mean RMSE errors for the whole region are shown in brackets (E) on top of each plot.

Figure 10 (Supplementary Fig. 8) illustrates a comparison between the RMSE errors in the projected annual maximum (minimum) temperature for the 2050 s under the SSP3-7.0 emission scenario, before and after applying downscaling techniques. As with the precipitation, the evaluation by the *perfect sibling* comparison showed that downscaling substantially decreased the bias (the mean RMSEs over the whole region are denoted by E measured in °C are shown on the top of each figure) in the projected annual temperature outputs (Fig. 10, Supplementary Fig. 8 & Supplementary Table 4). For maximum temperature, the minimum reduction in error was 0.9 °C (~70% lower) for EC-Earth3-Veg while the maximum reduction was 7.3 °C (~93% lower) for MIROC6 (Fig. 10). Whereas for minimum temperature, the minimum reduction in error was 0.8 °C (~60% lower) for GFDL-ESM4 while the maximum reduction was 4.2 °C (~93% lower) for CMCC-CM2-SR5 (Supplementary Fig. 8). Overall, the downscaling strategy reduced the errors in projected annual temperature approximately by about 51 to 94%. Using a simple delta bias correction strategy, comparable bias minimization was reported through the *perfect sibling* evaluation framework^2^. Our results confirm that better performance is found for temperature compared to precipitation in regions where climate systems are modulated by complex topography^84^.

Our downscaled data sets provide high-resolution climate projections that can be used to assess the potential impacts of climate change on various sectors such as agriculture, water resources, and energy. By using our data sets, researchers can gain valuable insights into the potential changes in climate patterns at a local or regional scale, which can inform decision-making and policy development. However, it is important to note that our data sets have some limitations that should be considered when interpreting the results of impact studies. For example, the downscaling technique used in this study assumes that the historic model bias is stationary and valid for the future while it is not warranted that this is the case. Additionally, we would like to note that the technique used here is merely a statistical post-processing and cannot compensate or eliminate the individual GCM’s structural deficiencies. Furthermore, these techniques are not always perfect and can introduce their own sources of bias, so it is important to carefully evaluate the quality of the data generated using these methods. Furthermore, the presented evaluation could only cover some central aspects and further evaluations, e.g., interannual variability or spell length distributions, are encouraged to help explain the uncertainties related to the methodology^85,86^. Therefore, we recommend that researchers carefully evaluate the assumptions and limitations of our data sets when interpreting the results of their impact studies. In terms of the downscaled data sets from the different models, we recommend using the data sets that have the highest spatial correlations and lowest bias and errors (see Supplementary Table 1), as these will likely provide the most accurate representation of the local climate. However, we encourage researchers to explore the performance of multiple downscaled data sets from different models to evaluate the uncertainty associated with the projections.

## Supplementary information

Supplementary Information

## Data Availability

The R packages used in this study are freely available and detailed in the methods section. The R codes utilized for the analysis, along with the necessary input data, are publicly available at 10.5281/zenodo.7950777. The code demonstrates how to implement the climate downscaling technique and compute the means and sums of climate variables for various periods on the netcdf files to replicate all the findings mentioned in the manuscript.
