# Supplementary material for: High-resolution CMIP6 climate projections for Ethiopia using the gridded statistical downscaling method
Source: Sci Data. 2023 Jul 12;10:442. doi: 10.1038/s41597-023-02337-2 (PMC10338452; doi:10.1038/s41597-023-02337-2)
Supplement: Supplementary file 1 — Supplementary Information [file 41597_2023_2337_MOESM1_ESM.pdf]

High-resolution CMIP6 climate projections for Ethiopia using the gridded statistical downscaling method

Authors

Fasil M. Rettie[1, 2*], Sebastian Gayler[1], Tobias KD Weber[1,3], Kindie Tesfaye[4], Thilo Streck[1]

Affiliations

[1]Biogeophysics, Institute of Soil Science and Land Evaluation, Hohenheim University, 70599, Stuttgart, Germany

[2]Ethiopian Institute of Agricultural Research (EIAR), Melkasa, Ethiopia

[3]Soil Science Section, Faculty of Organic Agricultural Sciences, University of Kassel

[4]International Maize and Wheat Improvement Center (CIMMYT), Addis Ababa, Ethiopia

corresponding author*: Fasil M. Rettie (fasil.mequanint@uni-hohenheim.de, fasil.mequanint@gmail.com)

Supplementary Information

Table S1. Summary statistics of the countrywide area-averaged quantiles of mean bias, RMSE, and Pearson correlation coefficient (r) for mean JJAS total precipitation for all GCMs compared with the observation data (1983-2012). 3

Table S2. Summary statistics of the countrywide area-averaged quantiles of mean bias, RMSE, and Pearson correlation coefficient (r) for mean annual minimum temperature for all GCMs compared with the observation data (1983-2012). 3

Table S3. Summary statistics of the countrywide area-averaged quantiles of mean bias, RMSE, and Pearson correlation coefficient (r) for mean annual maximum temperature for all GCMs compared with the observation data (1983-2012). 4

Table S4. Countrywide area-averaged RMSE of mean JJAS total precipitation (Pr) and mean annual maximum (Tmax) and minimum (Tmin) temperatures between of the GCMs compared with the selected perfect sibling model (MRI-ESM2-0) evaluated for the projected climate period (2050's). 4

Figure S1. Elevation map of Ethiopia (a), and spatial plots of JJAS total (mm) precipitation (b), annual averaged maximum (c) and minimum (d) temperatures (°C) based on observation data (1983-2012). 5

Figure S2. Spatial plots comparing downscaled (2050) JJAS total precipitation (a, d in mm), projected absolute changes (b, e, in mm), and percentage change (c, f) relative to the observation for the two models, CMCC-CM2-SR5 (1st row) and MPI-ESM1-2-HR (2nd row). 6

Figure S3. Spatial plots comparing downscaled (2050s) mean annual maximum temperature (a, c, in °C) and the respective projected changes (b, d, in °C) from the observation for the two models (i.e., CMCC-CM2-SR5, 1st row and MPI-ESM1-2-HR, 2nd row). 7

Figure S4. Spatial plots comparing historical (1983-2012) biases in mean annual minimum temperature (°C) before (a, c) and after (b, d) downscaling for the two models, CMCC-CM2-SR5 (1st row) and MPI-ESM1-2-HR (2nd row). The base map shows the nine homogeneous precipitation subregions in text (R1, R2, …, R9). 8

Figure S5. Spatial plots comparing downscaled (2050s) mean annual minimum temperature (a, c, in °C) and the respective projected changes (b, d, in °C) from the observation for the two models, CMCC-CM2-SR5 (1st row) and MPI-ESM1-2-HR (2nd row) in °C. 9

Figure S6. Cumulative density function plots for mean annual minimum temperature (°C) for the nine homogeneous precipitation subregions (R1, R2, …, R9) under SSP3-7.0 scenario. HR represents models' historical climate before downscaling and, HD and FD represent models' historical climate and future projections after downscaling. Obs represent the observation data for the base period. 10

Figure S7. Spatial distribution of correlation coefficient (left column) and RMSE (right column) between models and observation before (upper row) and after (lower row) downscaling for annual mean minimum temperature for the period 1983-2012. 11

Figure S8. Evaluation (based on RMSE) of GCM outputs using the PS framework (MRI-ESM2-0 selected as a perfect sibling) for annual mean minimum temperature for the 2050s under the SSP3-7.0 scenario, a) before downscaling and b) after downscaling. The mean RMSE errors for the whole region are shown in brackets (E) on top of each plot. 12

Table S1. Summary statistics of the countrywide area-averaged quantiles of mean bias, RMSE, and Pearson correlation coefficient (r) for mean JJAS total precipitation for all GCMs compared with the observation data (1983-2012).

	Before downscaling					After downscaling				
				Bias					Bias	
GCMs	RMSE	Pearson's r	q5	q50	q95	RMSE	Pearson's r	q5	q50	q95
ACCESS-CM2	59.0	0.49	-680	-256	10	55.6	0.66	-33	0	17
ACCESS-ESM1-5	78.0	0.48	-143	292	759	55.7	0.66	-35	11	62
CMCC-CM2-SR5	76.2	0.46	-360	3	205	67.1	0.33	-194	-47	35
GFDL-ESM4	76.9	0.41	-381	-15	171	64.9	0.35	-207	-64	47
INM-CM4-8	77.6	0.39	-360	139	498	68.7	0.33	-192	-49	44
INM-CM5-0	68.5	0.41	-464	-27	240	68.8	0.33	-206	-63	49
MIROC6	76.0	0.62	-86	400	1234	44.5	0.71	-5	11	39
MPI-ESM1-2-HR	56.1	0.60	-369	-179	144	55.3	0.65	-13	2	30
MPI-ESM1-2-LR	55.3	0.59	-407	-232	99	55.3	0.63	-28	-4	19
MRI-ESM2-0	73.1	0.53	-426	-82	574	53.4	0.65	-17	1	23
NorESM2-LM	91.5	-0.27	-764	-508	-362	107.6	-0.25	-1001	-581	-110
NorESM2-MM	99.1	-0.29	-813	-471	-230	100.2	-0.25	-1008	-583	-110

Table S2. Summary statistics of the countrywide area-averaged quantiles of mean bias, RMSE, and Pearson correlation coefficient (r) for mean annual minimum temperature for all GCMs compared with the observation data (1983-2012).

GCMs	Before downscaling					After downscaling				
				Bias					Bias	
	RMSE	Pearson's r	q5	q50	q95	RMSE	Pearson's r	q5	q50	q95
ACCESS-CM2	3.02	0.63	-4.749	-0.905	2.832	1.09	0.77	-0.008	-0.001	0.007
ACCESS-ESM1-5	3.31	0.58	-4.109	-1.104	1.439	1.08	0.76	-0.016	-0.004	0.007
AWI-CM-1-1-MR	2.53	0.66	-2.241	-0.061	2.384	1.13	0.75	-0.008	0.001	0.018
CMCC-CM2-SR5	5.74	0.15	1.288	4.702	8.864	1.75	0.49	-0.004	0.012	0.030
EC-Earth3	3.19	0.56	-4.867	-2.146	0.484	1.09	0.76	-0.010	-0.001	0.012
EC-Earth3-Veg	3.22	0.57	-4.8	-2.1	0.4	1.11	0.75	-0.008	0.000	0.011
GFDL-ESM4	3.08	0.47	-3.922	-1.371	0.841	1.69	0.52	0.013	0.027	0.052
INM-CM4-8	4.71	0.41	-6.967	-2.607	1.702	1.93	0.36	0.017	0.041	0.076
INM-CM5-0	4.67	0.41	-7.01	-2.713	2.003	1.94	0.35	0.010	0.034	0.073
IPSL-CM6A-LR	3.48	0.58	-4.409	-2.211	2.005	1.11	0.75	-0.004	0.001	0.008
MIROC6	3.67	0.55	-0.696	2.063	7.106	1.15	0.74	-0.023	-0.004	0.004
MPI-ESM1-2-HR	2.59	0.66	-2.183	0.229	2.665	1.10	0.75	-0.018	-0.001	0.010
MPI-ESM1-2-LR	3.02	0.63	-2.083	0.089	3.192	1.11	0.75	-0.013	-0.002	0.008
MRI-ESM2-0	2.39	0.64	-3.409	-0.325	1.998	1.07	0.76	-0.016	-0.006	0.003
NorESM2-LM	4.49	-0.26	-1.159	1.322	3.734	3.14	-0.30	0.000	0.034	0.071
NorESM2-MM	4.01	-0.29	-2.824	-0.11	1.775	3.16	-0.31	0.041	0.069	0.105

Table S3. Summary statistics of the countrywide area-averaged quantiles of mean bias, RMSE, and Pearson correlation coefficient (r) for mean annual maximum temperature for all GCMs compared with the observation data (1983-2012).

GCMs	Before downscaling					After downscaling				
			Bias					Bias		
	RMSE	Pearson's r	q5	q50	q95	RMSE	Pearson's r	q5	q50	q95
ACCESS-CM2	3.15	0.62	-5.448	-0.992	2.584	1.14	0.78	-0.012	-0.002	0.004
ACCESS-ESM1-5	3.72	0.64	-6.352	-1.802	0.187	1.13	0.79	-0.02	-0.003	0.012
AWI-CM-1-1-MR	2.71	0.73	-3.954	-0.95	0.801	1.2	0.77	-0.008	0.003	0.018
CMCC-CM2-SR5	6.39	0.49	-8.677	-5.961	-3.439	1.79	0.53	-0.008	0.005	0.018
EC-Earth3	2.7	0.64	-4.213	-0.97	0.885	1.16	0.78	-0.01	-0.001	0.01
EC-Earth3-Veg	2.77	0.63	-4.031	-0.91	0.89	1.24	0.75	-0.005	0.005	0.03
GFDL-ESM4	4.17	0.48	-5.365	-3.099	-1.368	1.78	0.53	-0.006	0.011	0.027
INM-CM4-8	4.22	0.34	-5.008	-2.604	2.103	1.74	0.54	-0.017	-0.002	0.024
INM-CM5-0	4.06	0.39	-5.036	-2.174	2.752	1.75	0.54	-0.016	0	0.013
IPSL-CM6A-LR	5.16	0.62	-6.881	-4.429	-2.302	1.23	0.76	-0.005	0.001	0.008
MIROC6	7.16	0.51	-2.046	5.864	15.284	1.2	0.77	-0.066	0.015	0.043
MPI-ESM1-2-HR	2.63	0.7	-3.203	-0.748	0.994	1.19	0.77	-0.017	0.002	0.012
MPI-ESM1-2-LR	3.2	0.72	-3.905	-1.57	0.742	1.15	0.78	-0.012	-0.003	0.007
MRI-ESM2-0	3.01	0.53	-4.202	-0.613	2.083	1.28	0.73	-0.057	-0.008	0.016
NorESM2-LM	4.54	-0.15	-3.834	-1.278	0.685	3.22	-0.29	-0.04	-0.011	0.039
NorESM2-MM	4.52	-0.23	-4.829	-2.214	0.413	3.24	-0.32	0.02	0.038	0.067

Table S4. Countrywide area-averaged RMSE of mean JJAS total precipitation (Pr) and mean annual maximum (Tmax) and minimum (Tmin) temperatures between of the GCMs compared with the selected perfect sibling model (MRI-ESM2-0) evaluated for the projected climate period (2050's). -- GCMs do not include precipitation data.

GCMs	Before downscaling			After downscaling			Error reduction (%)		
	Pr	Tmax	Tmin	Pr	Tmax	Tmin	Pr	Tmax	Tmin
ACCESS-CM2	308.3	2.46	1.79	182.9	0.94	0.87	40.7	61.8	51.5
ACCESS-ESM1-5	313.5	2.91	1.94	85.9	0.56	0.55	72.6	80.6	71.7
AWI-CM-1-1-MR	--	1.53	0.98	--	0.18	0.22	--	88.5	77.1
CMCC-CM2-SR5	420.4	5.99	4.83	158.6	0.37	0.68	62.3	93.8	85.9
EC-Earth3	--	1.26	1.67	--	0.35	0.59	--	72.0	64.5
EC-Earth3-Veg	--	1.21	1.79	--	0.22	0.47	--	81.6	73.8
GFDL-ESM4	291.3	3.12	1.36	139.2	0.34	0.56	52.2	89.0	59.1
INM-CM4-8	450.8	3.51	3.84	138.6	0.41	0.26	69.3	88.4	93.4
INM-CM5-0	359.2	3.37	3.89	121.5	0.45	0.32	66.2	86.5	91.8
IPSL-CM6A-LR	--	4.26	2.13	--	0.53	0.56	--	87.6	73.7
MIROC6	406.0	7.83	3.18	63.8	0.56	0.64	84.3	92.9	80.0
MPI-ESM1-2-HR	231.3	1.49	1.00	83.3	0.34	0.17	64.0	77.4	83.3
MPI-ESM1-2-LR	290.9	2.35	1.78	86.3	0.27	0.25	70.3	88.6	85.8
MRI-ESM2-0	0.0	0.00	0.00	0.0	0.00	0.	0.00	0.00	0.00
NorESM2-LM	449.8	2.69	2.80	450.8	0.27	0.22	-0.2	90.0	92.3

NorESM2-MM	446.7	2.05	1.26	449.6	0.47	0.31	-0.6	77.2	75.5

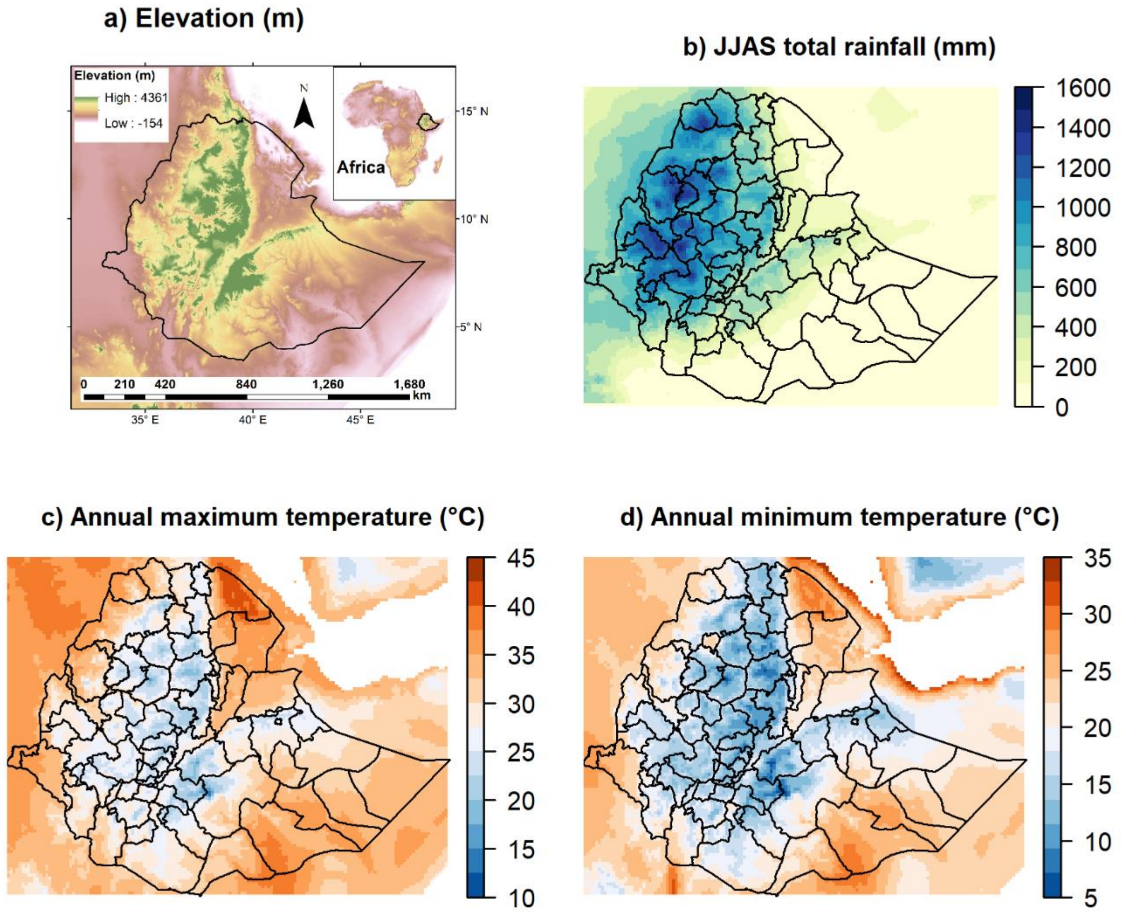

Figure S1. Elevation map of Ethiopia (a), and spatial plots of JJAS total precipitation (b), annual averaged maximum temperature (c) and minimum temperature (d) based on observation data (1983-2012).

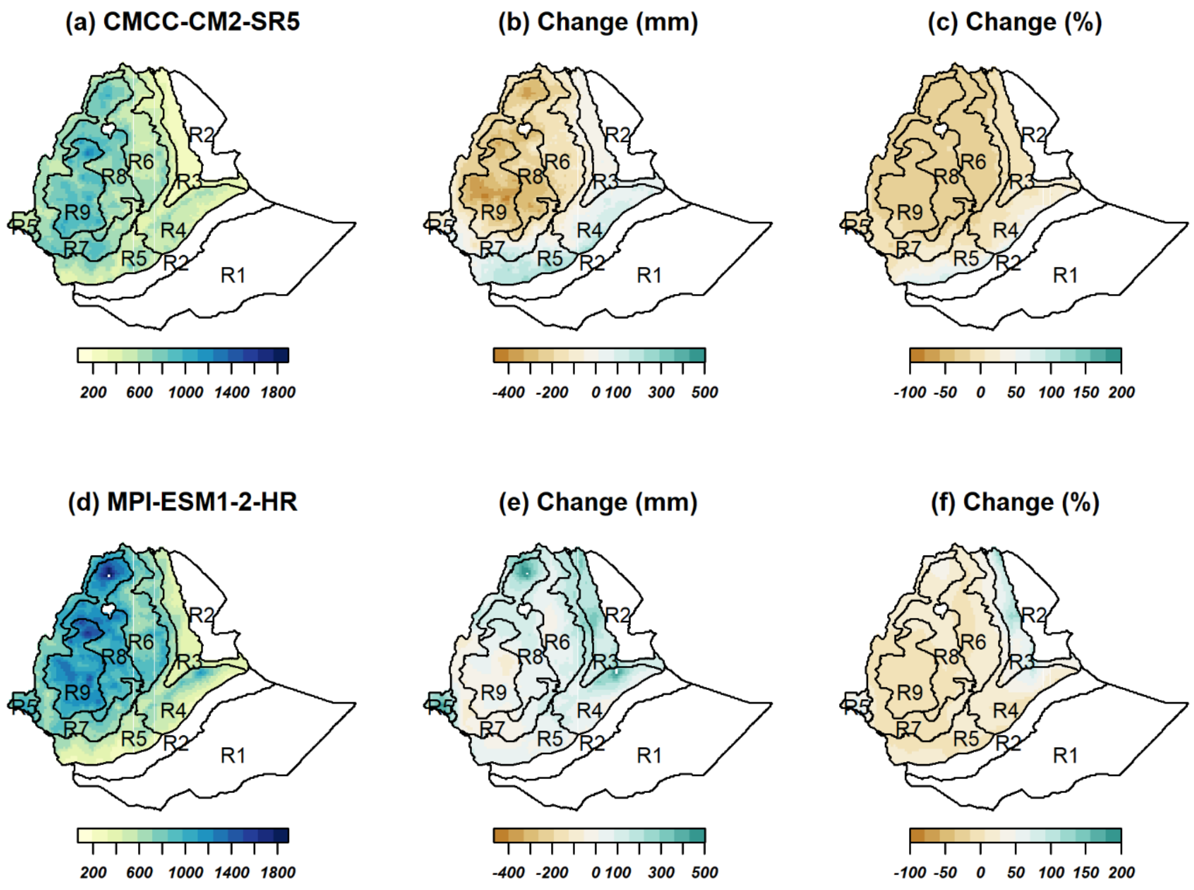

Figure S2. Spatial plots comparing downscaled (2050) JJAS total precipitation (a, d in mm), projected absolute changes (b, e, in mm), and percentage change (c, f) relative to the observation for the two models, CMCC-CM2-SR5 (1st row) and MPI-ESM1-2-HR (2nd row).

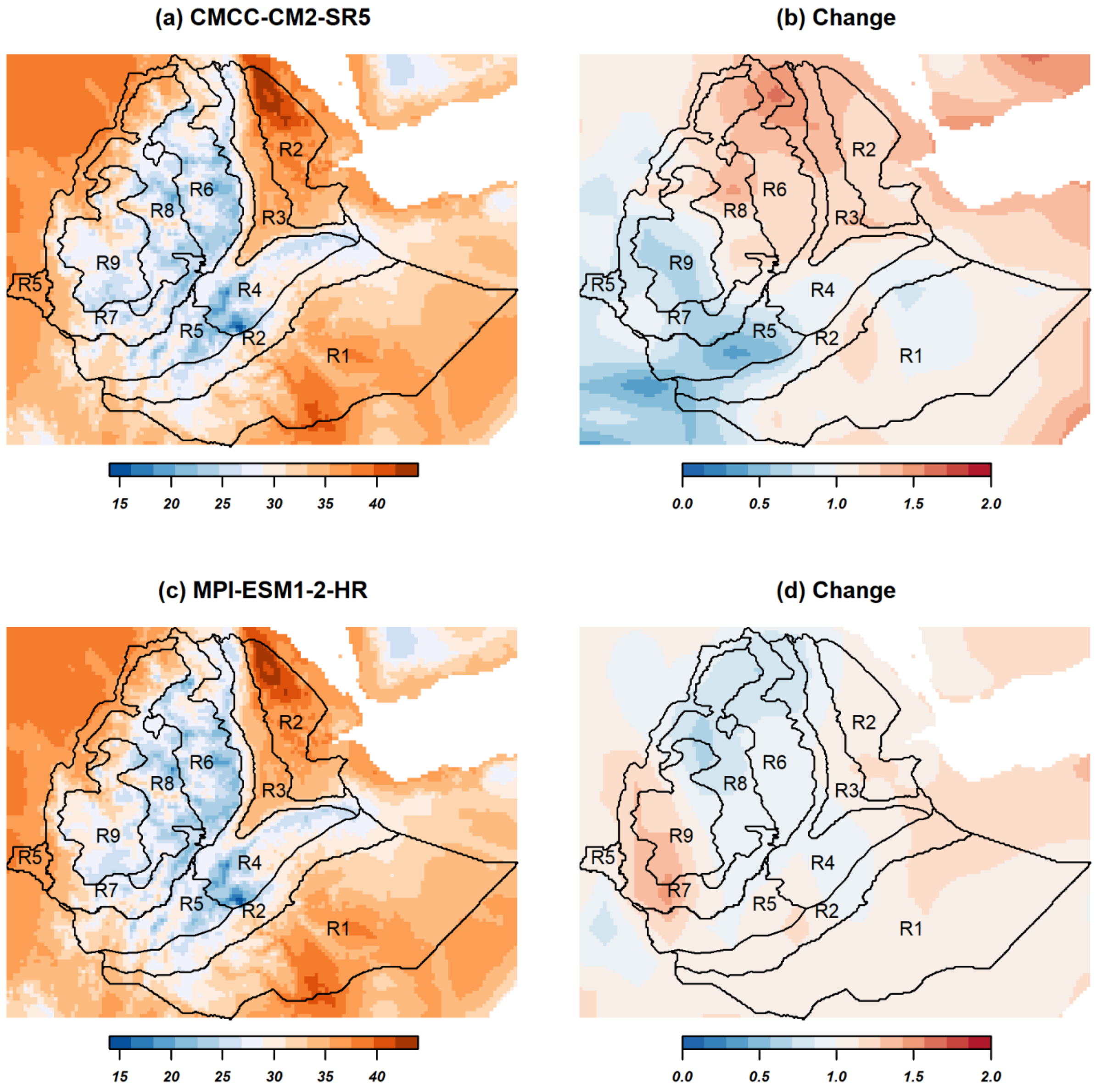

Figure S3. Spatial plots comparing downscaled (2050s) mean annual maximum temperature (a, c, in °C) and the respective projected changes (b, d, in °C) from the observation for the two models (i.e., CMCC-CM2-SR5, 1st row and MPI-ESM1-2-HR, 2nd row).

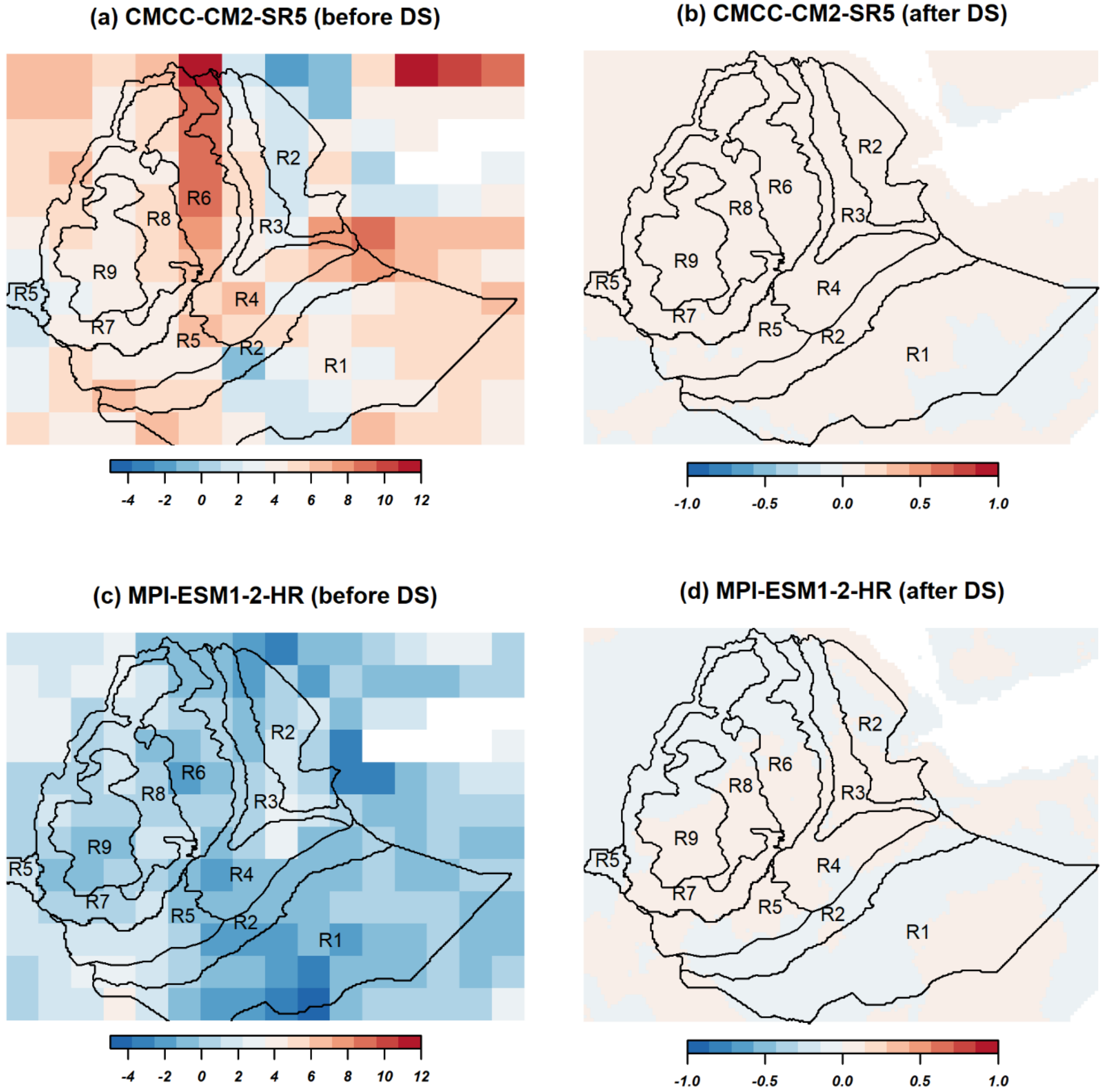

Figure S4. Spatial plots comparing historical (1983-2012) biases in mean annual minimum temperature (°C) before (a, c) and after (b, d) downscaling for the two models, CMCC-CM2-SR5 (1st row) and MPI-ESM1-2-HR (2nd row). The base map shows the nine homogeneous precipitation subregions in text (R1, R2, …, R9).

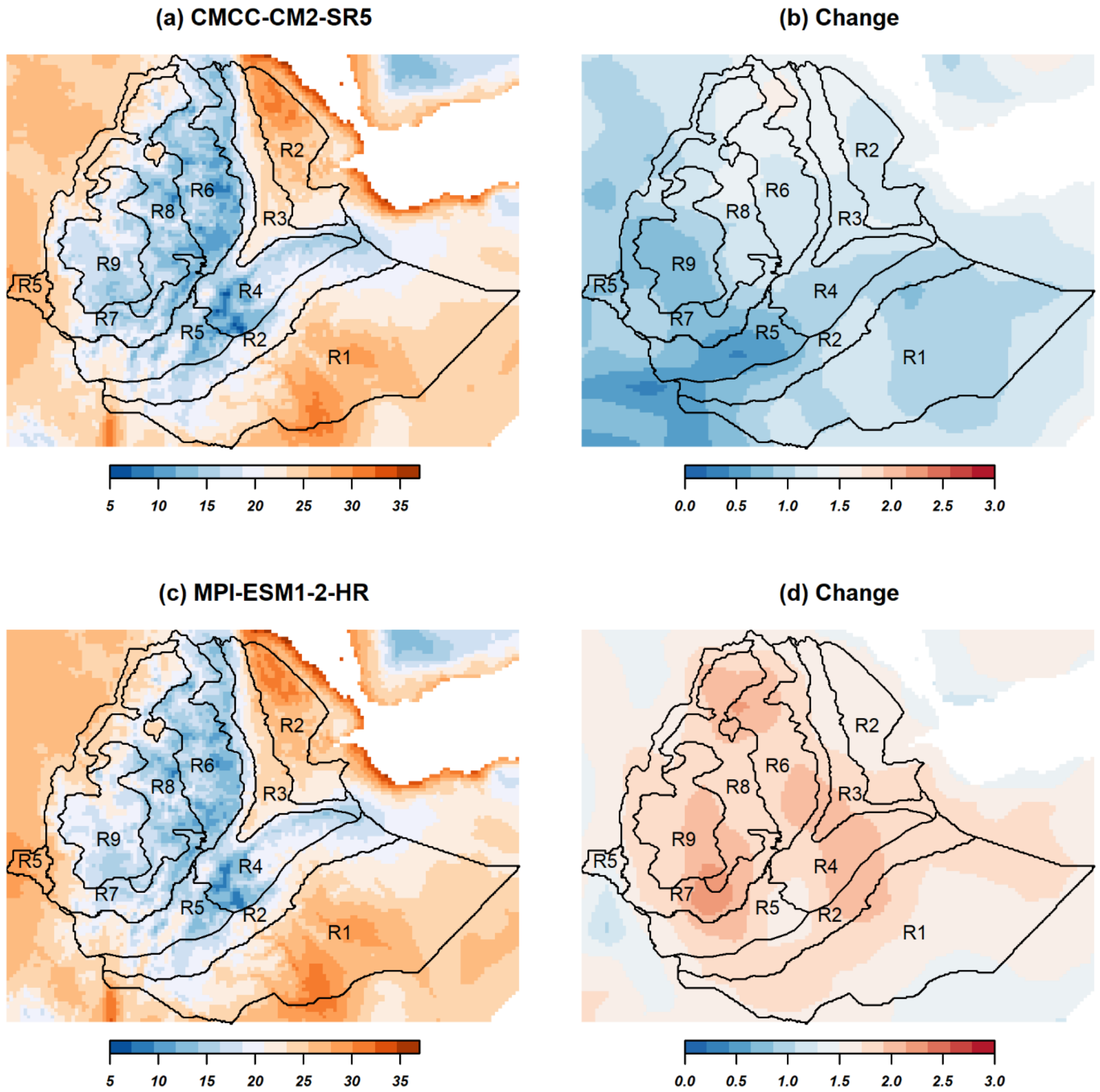

Figure S5. Spatial plots comparing downscaled (2050s) mean annual minimum temperature (a, c, in °C) and the respective projected changes (b, d, in °C) from the observation for the two models, CMCC-CM2-SR5 (1st row) and MPI-ESM1-2-HR (2nd row) in °C.

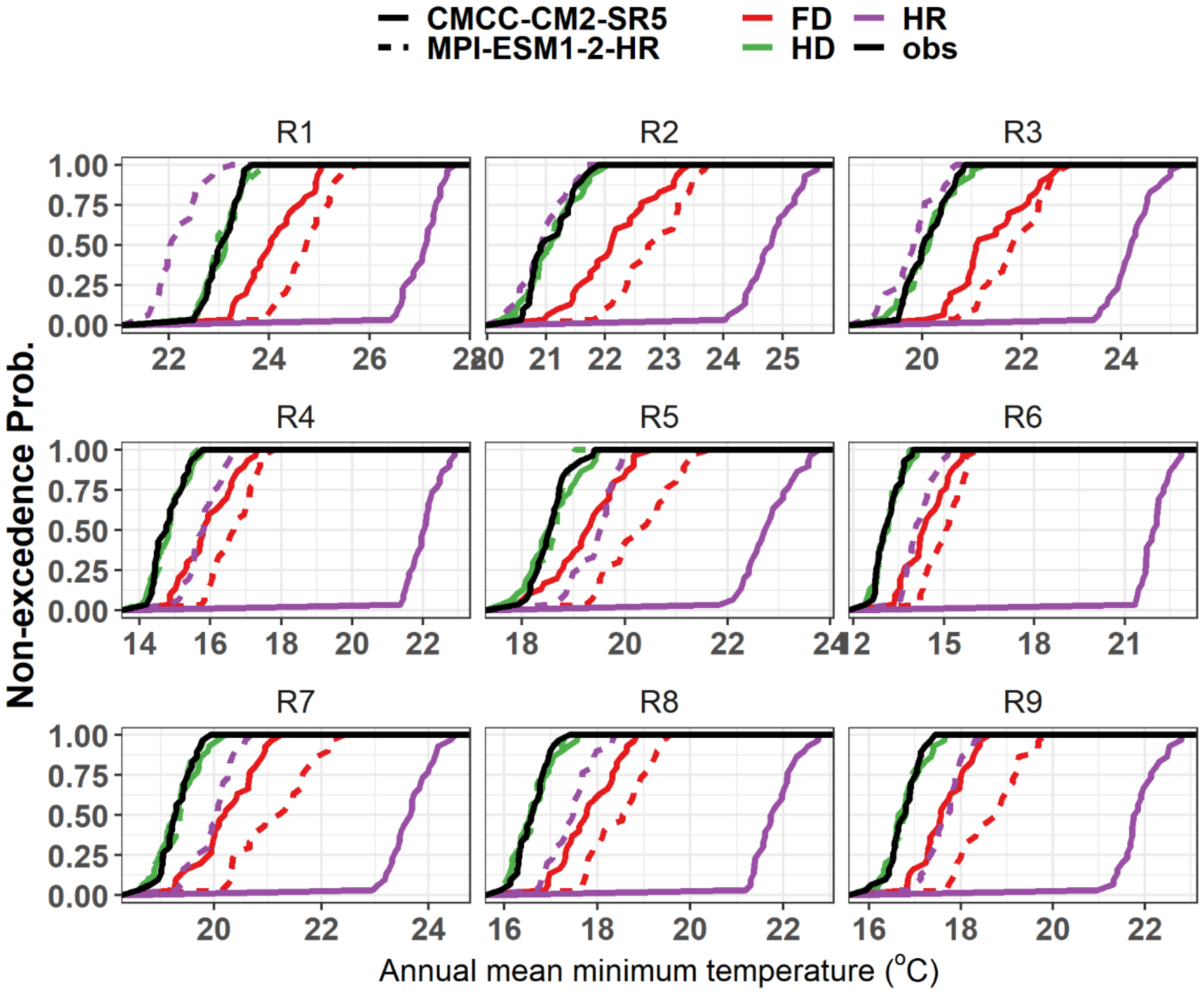

Figure S6. Cumulative density function plots for mean annual minimum temperature (°C) for the nine homogeneous precipitation subregions (R1, R2, …, R9) under SSP3-7.0 scenario. HR represents models' historical climate before downscaling and, HD and FD represent models' historical climate and future projections after downscaling. Obs represent the observation data for the base period.

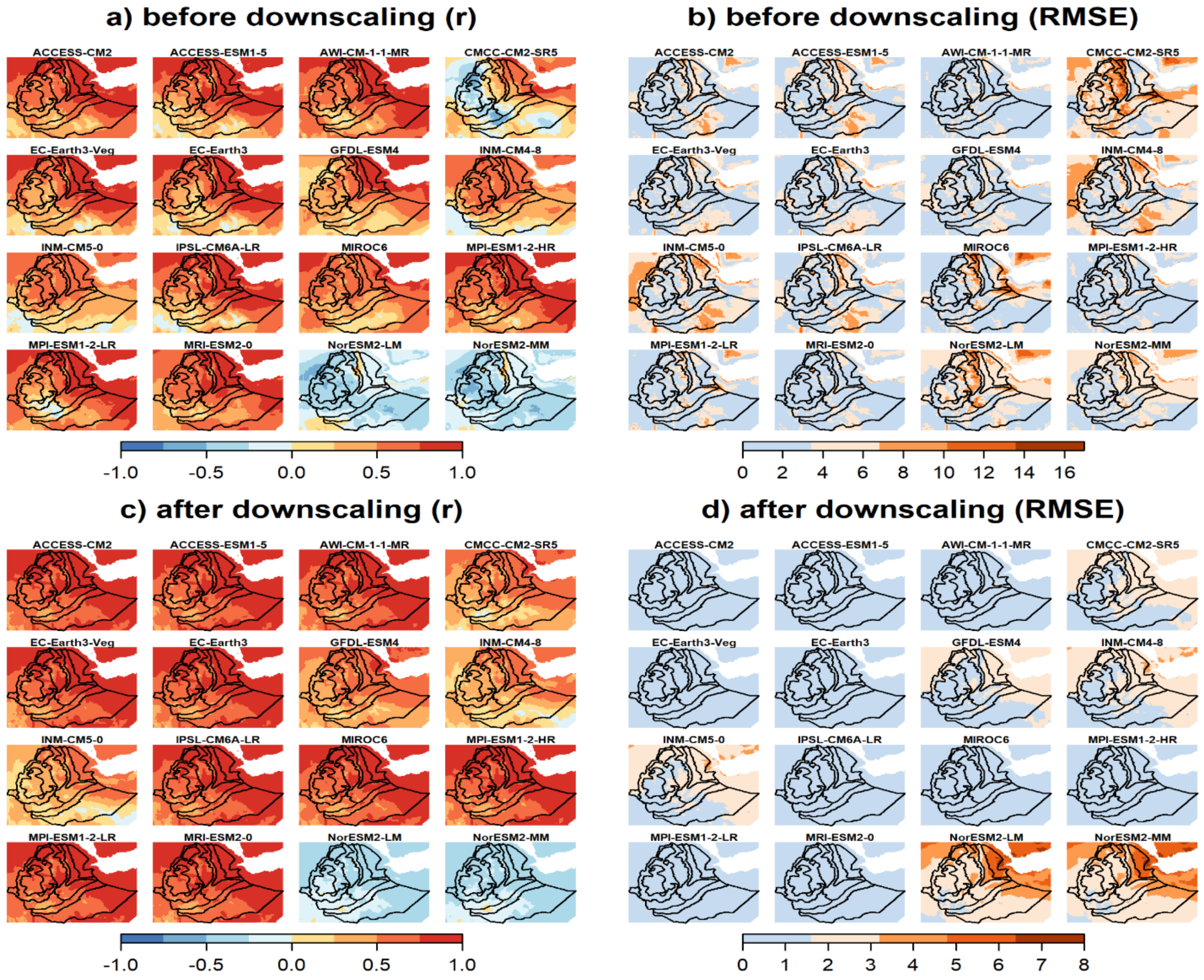

Figure S7. Spatial distribution of correlation coefficient (left column) and RMSE (right column) between models and observation before (upper row) and after (lower row) downscaling for annual mean minimum temperature for the period 1983-2012.

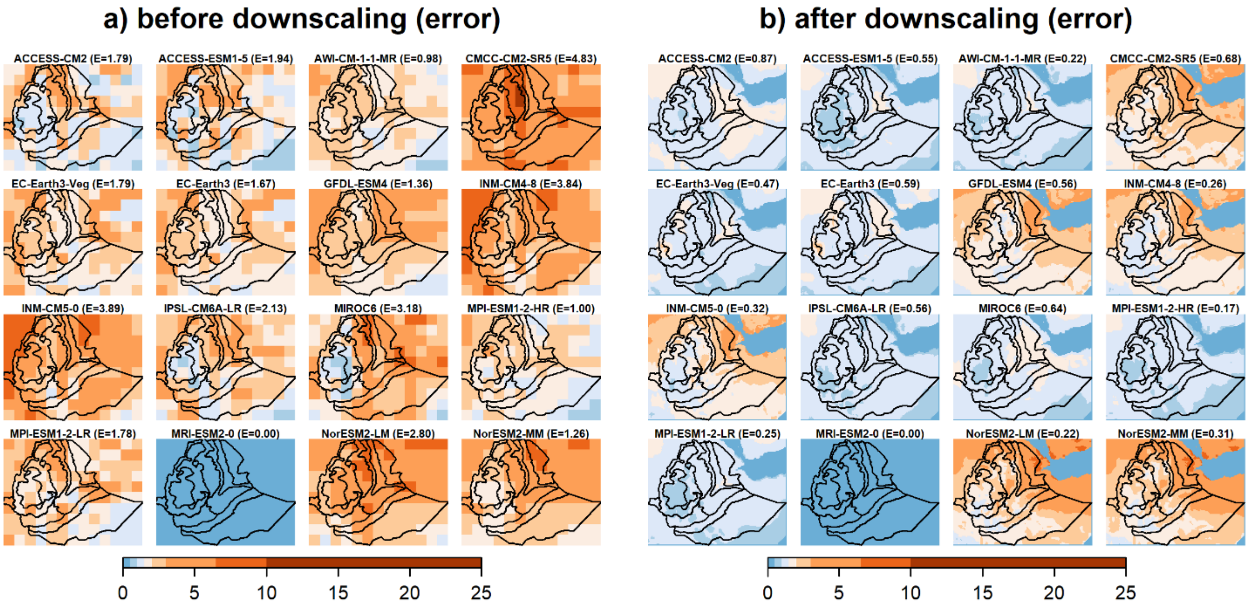

Figure S8. Evaluation (based on RMSE) of GCM outputs using the PS framework (MRI-ESM2-0 selected as a perfect sibling) for annual mean minimum temperature for the 2050s under the SSP3-7.0 scenario, a) before downscaling and b) after downscaling. The mean RMSE errors for the whole region are shown in brackets (E) on top of each plot.
